# Post‐translational Modifications in Proteins: Prediction Methods, Biological Functions, and Diseases

**DOI:** 10.1002/mco2.70729

**Published:** 2026-04-12

**Authors:** Shuning Zhang, Jingmin Li, Meihuan Chen, Hailong Huang

**Affiliations:** ^1^ Medical Genetic Diagnosis and Therapy Center of Fujian Maternity and Child Health Hospital College of Clinical Medicine for Obstetrics & Gynecology and Pediatrics Fujian Medical University Fuzhou China; ^2^ Fujian Provincial Key Laboratory for Prenatal Diagnosis and Birth Defect Fuzhou China

**Keywords:** clinical translation, erythroid disorders, mass spectrometry, posttranslational modifications, signaling cascades

## Abstract

Posttranslational modifications (PTMs) act as rapid, reversible switches that reshape protein activity, stability, and interactome, thereby governing virtually every physiological cue from signal transduction to epigenetic memory. Mass spectrometry‐based proteomics has considerably extended our knowledge about the occurrence and dynamics of PTMs. Pinpointing disease‐ or physiology‐specific PTM sites remains an open challenge. As a biological process with well‐defined stage‐specific markers and a precise endpoint, erythropoiesis is orchestrated by the complex interplay of multiple PTM‐regulatory networks, making it an ideal model for dissecting the spatiotemporal dynamics, quantitative thresholds, and crosstalk of PTMs. This review delineates the applications, detection, and prediction technologies of PTMs, with an emphasis on the mechanisms of phosphorylation, ubiquitination, methylation, SUMOylation, glycosylation, and acetylation in both physiological and pathological processes. Dissecting PTM circuitry driving erythroid specification and maturation, we show how its perturbation triggers disease, clarifying PTM roles. Additionally, we have investigated the progress made in the clinical translation and drug development of the PTMs field, emphasizing the potential of PTMs in the field of precision medicine as well as the existing challenges. This review aims to provide new insights and perspectives for the study of PTMs.

## Graphical abstract

Posttranslational modifications (PTMs) serve as rapid and reversible molecular switches that reshape protein activity, stability, and interactomes, thereby governing virtually all physiological cues ranging from signal transduction to epigenetic memory. Mass spectrometry‐based proteomics has substantially expanded our understanding of PTM emergence and dynamics. This review focuses on PTM detection and prediction technologies, as well as their clinical translation, with particular emphasis on the mechanisms of phosphorylation, ubiquitination, methylation, SUMOylation, glycosylation, and acetylation in physiological and pathological processes. We highlight the potential of PTMs in precision medicine and address current challenges in this field.

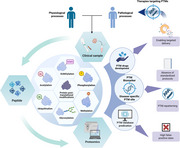

## 1 Introduction

Proteins are typically composed of only 20 canonical amino acids, yet their accessible chemical space is vastly expanded in living systems through posttranslational modifications (PTMs) of various amino‐acid side chains. PTMs are executed by dedicated enzymes that recognize either short linear motifs or three‐dimensional structural determinants on substrate proteins, ensuring precise residue selection and steric accessibility. These covalent marks are highly dynamic and can install or remove the adduct within seconds to minutes, enabling rapid cellular adaptation to external stimuli. Chemical incorporation alters local electrostatics or steric bulk, provoking conformational shifts, interface remodeling, or the creation of new binding surfaces [1]. Consequently, PTMs switch catalytic activity, redirect subcellular trafficking, remodel multiprotein complexes, and modulate gene expression. Single modifications can seed hierarchical cascades reprogramming, and stress responses, or compete with alternative marks on identical or adjacent residues, generating combinatorial regulatory states.

To date, more than 680 types of PTMs have been identified. These PTMs orchestrate gene transcription, intracellular and extracellular signaling cascades, as well as protein size, activity, stability, subcellular localization, trafficking, secretion, intracellular degradation or half‐life, and protein–protein interactions (PPIs) [2, 3, 4]. Aberrant PTMs frequently underlie pathogenesis; therefore, mapping disease‐associated PTMs not only deepens our understanding of the underlying molecular mechanisms but also enables the discovery of novel biomarkers for disease progression and prognosis, facilitates the development of personalized therapies, and identifies innovative drug targets.

The production process of red blood cells (RBCs), known as erythropoiesis, involves several successive stages that transform hematopoietic stem cells (HSCs) into mature RBCs. Erythropoiesis can be divided into three main stages: erythroid progenitor cells, erythroid precursor cells, and mature RBCs [5]. The entire erythropoiesis process, from the establishment of the erythroid lineage, the generation of erythroid progenitor cells to the terminal differentiation of RBC, along with the crucial conversion of hemoglobin, is orchestrated by a complex regulatory network (Figure S1). PTM, as the executor of fundamental cell‐identity switching, orchestrates organelle clearance, cytoskeletal reorganization, and functional specialization through an extreme and synchronized proteome remodeling during erythropoiesis. Perturbation of these PTMs is directly linked to major blood disorders, including hemoglobinopathies, congenital anemias, and myelodysplastic syndromes. Consequently, erythropoiesis serves as a unique bridge between basic modification mechanisms and human pathology. Moreover, the highly synchronous nature of red‐cell development, its well‐defined stage‐specific markers, and its unequivocal biological end‐point render it an ideal model for dissecting the temporal dynamics of PTMs, illuminating how they cooperate, relay, or antagonize one another in complex crosstalk. The principles gleaned from this system provide universal insights into pathological processes in other tissues. Driven by advances in detection technologies and the demand for more precise therapeutic targets in precision medicine, the pivotal roles of PTMs in regulating erythropoiesis and red‐cell diseases have been thoroughly investigated in recent years.

This review provides a comprehensive overview of PTM prediction methods, biological functions, and disease associations. We focus on PTM events, including phosphorylation, ubiquitination, methylation, SUMOylation, glycosylation, and acetylation, with the application of various PTM modifiers and the opportunities and challenges they present. Using erythropoiesis and the molecular pathogenesis of erythroid disorders as a clinically relevant paradigm, we specifically illustrate how individual PTMs operate and how they can be translated into therapeutic strategies.

## 2 Tools for PTM Identification and Prediction

**FIGURE 1:**
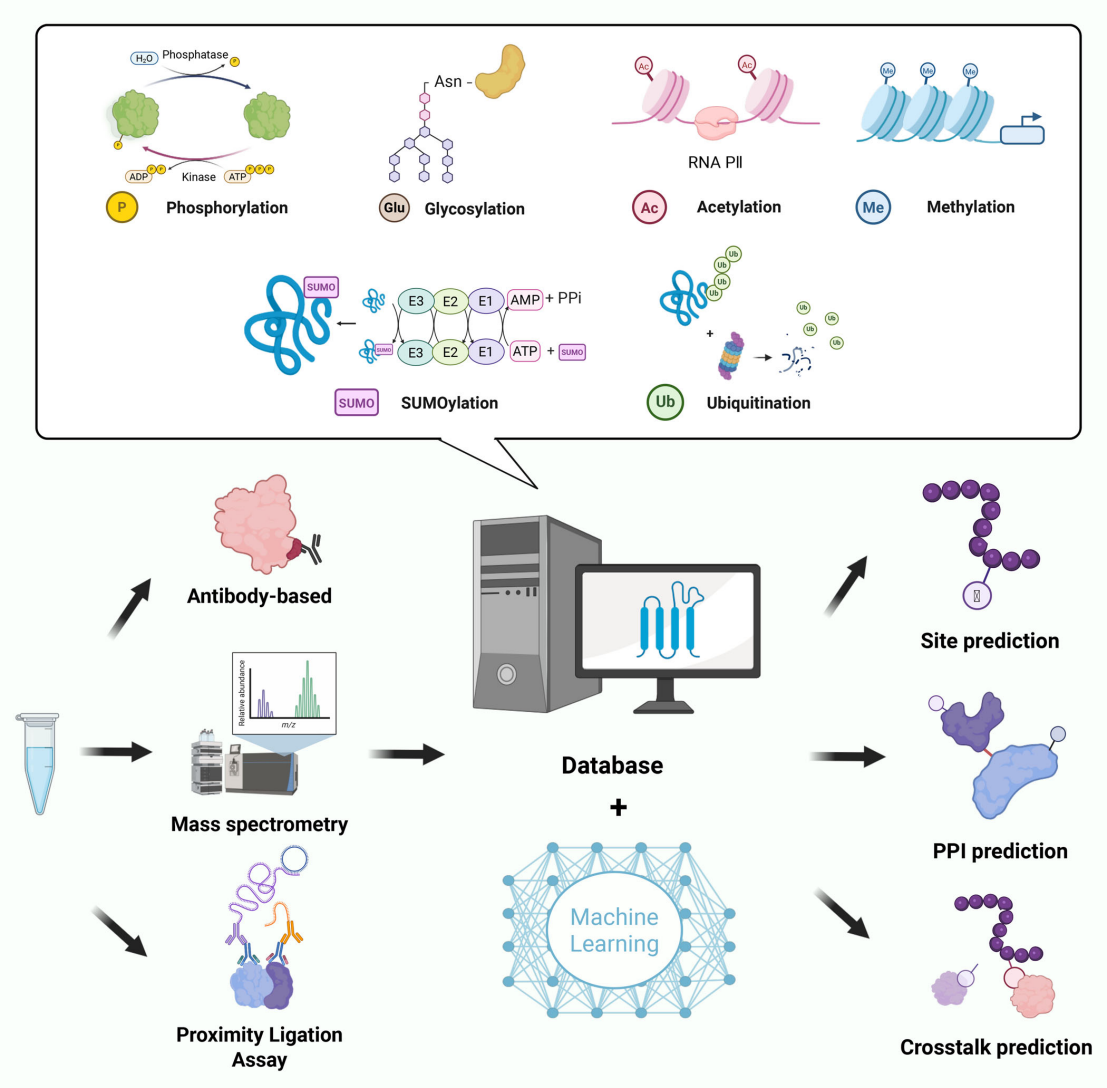
Tools for the identification and prediction of protein PTMs. Protein PTM identification is primarily carried out by mass spectrometry, specific antibodies, proximity ligation assays, and other approaches. The rapid accumulation of large‐scale PTM proteomic datasets now makes it possible to integrate the information in interactive databases. Bioinformatics tools complement experimental data through PTM site prediction, crosstalk analysis, and PPI prediction, increasingly powered by machine learning algorithms. Integrated use of these tools enhances the understanding of PTM networks and their biological roles. *Abbreviation*: PPI, protein–protein interaction.

Although over 600 distinct PTM classes have been identified, the chemistry of these marks is exquisitely subtle: they are often substoichiometric, transient, and mutually exclusive within the same peptide, while the enzymes that add and remove them operate in dynamic, context‐dependent networks that are rewired by cell type, metabolic state, and even sub‐organelle microenvironment. Therefore, predicting and detecting PTM sites remains an outstanding challenge in biochemistry and biology. With the rapid advancement of mass spectrometry (MS), thousands of novel modification sites have been identified. However, the high cost and the low throughput of experimental validation have become bottlenecks limiting research progress. Moreover, many modifications occur within the internal structures of proteins, making them inaccessible to traditional experimental approaches. The systematic integration of computational prediction and experimental identification is revolutionizing the landscape of PTM research. The following sections will introduce common PTM research methodologies and PTM prediction approaches (Figure 1).

### 2.1 PTM Identification Tools

#### 2.1.1 MS‐Based Approaches

MS is one of the most commonly used and powerful tools in proteomic studies for identifying PTM sites [6]. The typical workflow for PTM analysis is a bottom‐up approach, which includes extracting proteins from biological samples, digesting protein mixtures with proteases, enriching and/or fractionating peptides, and separating peptides by liquid chromatography before analysis in a mass spectrometer. During MS analysis, peptides are fragmented using collision‐induced dissociation or higher‐energy collisional dissociation for peptide sequencing and assignment of PTMs to specific sites [7, 8]. However, most PTMs are low‐abundance, unstable, and heterogeneous. Therefore, it is necessary to enrich modified peptides so that they can be identified and quantified by MS from complex samples such as cell lysates and tissue extracts. Top‐down proteomics, which bypasses enzymatic digestion and analyzes intact full‐length proteins, provides the most straightforward approach to accurate protein identification [9]. This approach can detect and quantify combinatorial PTM patterns on a single protein molecule, avoiding the loss of modification sites or the introduction of artificial chemical alterations that often accompany proteolytic digestion. However, it demands high protein purity (to minimize isoform heterogeneity) and offers lower sensitivity, making it best suited for low‐throughput, high‐complexity analyses.

In short, top‐down proteomics provides a “panoramic” view of the complete modification landscape of individual proteins, whereas bottom‐up proteomics reassembles a proteome‐wide modification map “piece‐by‐piece” from peptides. When applied complementarily, these approaches address biological questions ranging from single‐molecule PTM combinations to system‐level modification networks.

#### 2.1.2 Antibody‐Based Approaches

Using PTM‐specific antibodies to recognize modified proteins, combined with electrophoretic separation and assessment of signal intensity or mobility shifts, remains the mainstay of PTM research. These antibodies are also routinely employed in coimmunoprecipitation to analyze PTMs of target proteins [10].

Bryan Iii et al. validated and characterized several novel monoclonal antibodies targeting acetylated K280 and K311 residues of tau. These reagents are suitable for deciphering the complex PTM “code” of tauopathies, including Alzheimer's disease (AD), and provide new tools for biomarker detection and the development of anti‐tau therapeutics [11]. Burt et al. developed a novel cocktail of anti‐O‐GlcNAcylation monoclonal antibodies for immunoprecipitating endogenous O‐GlcNAcylation‐modified peptides from cells and tissues. Applying this enrichment strategy to synaptosomes from mouse brain, they identified more than 1300 unique O‐GlcNAcylation peptides and over 1000 modification sites [12].

It should be noted, however, that such approaches are restricted to the analysis of individual PTMs and provide no information about their abundance or stoichiometry. Moreover, the stringent specificity required for PTM‐specific antibodies carries a risk of cross‐reactivity, which may produce false‐positive results and lead to incorrect conclusions about the biological and pathological roles of these modifications [13, 14].

#### 2.1.3 Proximity Ligation Assay‐Based Approaches

Proximity ligation assay (PLA) is likewise an in situ, highly sensitive, and highly specific method for detecting PTMs [15]. By combining antibody specificity with PCR‐based amplification, PLA enables the detection of endogenously modified proteins in fixed cells or tissues at single‐molecule sensitivity, serving as an efficient bridge between omics discovery and functional validation [16].

The assay employs two pairs of antibodies, each conjugated to a distinct oligonucleotide sequence. When both antibody pairs bind specifically to the target protein complex and are brought into close proximity (<40 nm), the attached oligonucleotides are likewise juxtaposed [17]. A DNA ligase then joins the adjacent oligonucleotides to form an intact, amplifiable DNA circle. Rolling circle amplification is subsequently used to generate a long, single‐stranded DNA molecule containing multiple tandem repeats. Fluorescently labeled complementary oligonucleotide probes are hybridized to this amplified DNA. The resulting fluorescent signal, detectable by fluorescence microscopy or other imaging systems, is proportional to the abundance of the target protein complex, enabling both qualitative and quantitative analysis. This method enables specific and highly sensitive detection. When coupled with multiplex coexpression assays, it allows for mapping the subcellular localization of modified proteins and tracking the dynamics of these pools over time. This approach is now being used to detect and quantify PTM‐derived biomarkers in human diseases [16, 18, 19]. However, when PLA is employed to interrogate biological events, it is essential to benchmark antibody performance: both a PTM‐specific and a pan‐protein antibody must be supplied, and epitope masking or poor antibody pairing can readily generate false negatives [20].

### 2.2 PTM Prediction Tools

#### 2.2.1 Methods for PTM Site Prediction

Due to the considerable cost and difficulties of experimental methods for identifying PTMs, machine learning (ML) approaches have been developed based on large‐scale proteomics data to identify patterns, classify proteins, and predict PTM sites [21]. Predictor construction typically involves multiple steps, including data collection, feature extraction, model training, and performance evaluation [22]. Some commonly used algorithms are support vector machine, Bayesian decision theory, random forest (RF), and logistic regression [23, 24]. Traditional ML algorithms often require manual feature selection and are sensitive to the choice of parameters. In recent popular deep learning (DL) architectures, convolutional neural networks, recurrent neural networks, and long short‐term memory networks are capable of directly predicting PTM sites from raw input sequences without the need for any feature extraction steps. However, they require a large amount of labeled data for training and consume substantial computational resources. Commonly used PTM prediction tools include the following: phosphorylation (NetPhos [25], DeepPhospho [26], TransPhos [27], GPS [28], PhosphoPredict [29]), acetylation (DeepAcet [30], ProAcePred [31], GPS‐PAIL [32]), methylation (DeepPRMS [33], DeepRMethylSite [34]), ubiquitination (UbPred [35], DeepUbi [36]), glycosylation (DeepNGlyPred [37], LMNglyPred [38], Stack‐OglyPred‐PLM [39], SPRINT‐Gly [40]), SUMOylation (PSSM‐Sumo [41], GPS‐SUMO [42]). Owing to the “black‐box” nature of DL algorithms, the dynamic changes of modification sites, and the challenges associated with predicting complex modification types, typical PTM prediction models lack sufficient interpretability. Future research needs to further integrate multisource data, develop more efficient algorithms, and enhance the interpretability and reliability of models to promote the development and application of PTM prediction technologies.

#### 2.2.2 Methods for Predicting PTM Crosstalk

PTM crosstalk events play critical roles in biological processes. Many PTM sites from the same (intra) or different (inter) proteins often cooperate with each other to perform a function, which is defined as PTM crosstalk. Several ML methods have been developed to identify PTM crosstalk within proteins, but the accuracy is still far from satisfactory.

PTM‐X is the first publicly released tool for predicting PTM crosstalk; it determines whether any two PTM sites—within the same protein or across different proteins—engage in crosstalk without distinguishing the type of interaction [43]. PCTpred and DeepPCT constitute an iterative pair of models for predicting PTM crosstalk within proteins [44, 45]. The positive samples for both training and testing are directly inherited from the original PTM‐X dataset, with a small amount of additional data added. The former first systematically incorporated experimental 3D structures, employing 26 hand‐crafted residue‐ and residue‐pair‐level features within a RF framework to raise the training‐set AUC to ∼0.90 and demonstrated that structural information can mitigate distance bias. The latter completely upgrades to a DL paradigm, replacing experimental structures with AlphaFold2/3‐predicted ones, and builds a “Transformer+GNN+RF” tri‐module fusion system leveraging ESM‐2 sequence embeddings, GearNet‐Edge graph networks, and novel geometric–topological descriptors. It reaches an AUC of 0.957 on the same training set and 0.777 on an independent test set, achieves a 60‐fold speed‐up in inference, and for the first time remains robust under strictly distance‐bias‐free conditions, thus spanning from reliance on experimental structures to proteome‐wide universality. Zhu et al. propose a novel integrated deep neural network PPICT (Predictor for PTM Inter‐protein Cross‐Talk), which predicts PTM crosstalk by combining protein sequence–structure–dynamics information and structural information for PPI graph [46]. PPICT introduces additional context at the PPI‐network level, capturing higher‐order associations across proteins or sites through multiscale embeddings and bilinear fusion, whereas PCTpred and DeepPCT focus solely on intraprotein crosstalk and rely on handcrafted features or deep pretraining strategies, respectively.

Although current predicting models for PTM crosstalk have greatly improved computational efficiency and scalability, they remain constrained by the scarcity of experimentally validated data, which leads to severe positive/negative sample imbalance, limited cross‐context generalization, and an inability to predict crosstalk events that depend on protein complex membership or subcellular localization. Future work must therefore continue to refine these models along the above directions.

#### 2.2.3 Tools for Analyzing PTM‐Associated PPIs

In living systems, PTMs of amino‐acid residues endow proteins with expanded chemical space and functional repertoires. These marks act as molecular beacons that recruit reader proteins to the modified sites, while also reshaping interaction networks indirectly‐by altering conformation or subcellular localization. Mapping the distinctive PPI networks triggered by each PTM is therefore essential for fully grasping the biological impact of this ever‐growing modification catalog [47].

iPTMnet employs automatic text mining to integrate, across multiple databases, published experimental evidence on PTMs (covering kinase–substrate relationships, modification sites, interactions, and regulatory links) and supplements this with interactive visualization to construct PTM networks [48]. PTMint is the first curated compendium of experimentally verified PTM effects on PPIs. It assembles full experimental details (including PTM type and position, binding partners, detection assays, linked diseases, and colocalization data) and systematically annotates each record with corresponding sequence and structural information, including molecular docking and interaction analyses [49]. Moreover, the website integrates PhosPPI, a sequence‐based machine‐learning method designed to predict the impact of phosphorylation on PPIs [50].

Despite the exponential growth in data and algorithms, PTM‐driven PPI prediction and integration still face the same fundamental bottlenecks that plague other predicting disciplines: scarce high‐quality negative samples, limited cross‐context generalizability, and the difficulty of establishing causal mechanisms.

## 3 Biological Functions of PTMs

PTMs precisely modulate protein structure, conformation, and physicochemical properties by influencing protein folding, stability, cellular localization, catalytic activity, and interactions with other proteins or biomolecules, thereby ultimately regulating key biological pathways such as signal transduction, protein trafficking and storage, gene expression, and intermolecular binding and affinity. Several modifications are physiologically more prevalent and have consequently been extensively studied. Remarkable advances in PTM‐enrichment chemistries and MS‐based workflows have expanded the catalog of confidently mapped modification sites to over 260,000 residues; however, the biological relevance of the vast majority of these sites remains to be fully elucidated [51].

Here, we focus on the fundamental regulatory mechanisms governing phosphorylation, ubiquitination, SUMOylation, glycosylation, and the acetylation and methylation of histones, and we illustrate their roles in detail using erythropoiesis as a model system.

### 3.1 Universal Regulatory Mechanisms

#### 3.1.1 Phosphorylation Modification

Protein phosphorylation is one of the most abundant PTMs in humans and is involved in regulating numerous physiological processes [52]. The protein phosphorylation system consists of kinases, phosphatases, phosphorylation substrates, and phosphorylation‐binding proteins [53]. This system functions by adding a phosphate group from adenosine triphosphate (ATP) to the side chains of amino acids through the action of kinases. The phosphorylation events act as molecular switches that can turn proteins on or off, alter their localization, or change their interactions with other cellular components. Phosphorylation of proteins occurs predominantly on Tyr, Ser, and Thr residues. There are approximately 500 protein kinases in the human genome, with approximately 90 being specific for Tyr residues, whereas the remaining 400+ kinases phosphorylate Ser/Thr residues [54]. DNA replication and mitosis are dependent on the activity of cyclin‐dependent protein kinase (CDK) enzymes for example. The mitogen‐activated protein kinase (MAPK) cascade constitutes a central intracellular signaling pathway. Each MAPK signaling cascade comprises three to five tiers of protein kinases that are sequentially activated by phosphorylation. Protein phosphorylation enables rapid and precise regulation of life processes through a reversible chemical modification, serving as a central mechanism in cellular signal transduction, metabolic adaptation, and disease pathogenesis.

#### 3.1.2 Ubiquitination Modifications

Ubiquitin (Ub) is a highly conserved polypeptide consisting of 76 amino acids that is widely expressed in eukaryotic cells [55]. Ubiquitination denotes the process by which Ub is covalently attached to target proteins via a cascade of specialized enzymatic reactions. A hallmark of Ub is the presence of seven lysine (Lys) residues, all of which can serve as acceptors for further ubiquitination, thereby generating isopeptide‐linked Ub chains. The covalent conjugation of Ub is orchestrated by a highly ordered ATP‐dependent enzymatic triad: E1 (Ub‐activating enzyme), E2 (Ub‐conjugating enzyme), and E3 (Ub ligase). E1 catalyzes the ATP‐dependent formation of a high‐energy thioester between the C‐terminal Gly of Ub and its catalytic cysteine. The activated Ub is subsequently trans‐thioesterified to a cysteine within the E2 active site. Substrate specificity is determined by E3 ligases, which mainly belong to the RING or HECT families. E3 ligases facilitate the nucleophilic attack of the ε‐amino group of a Lys residue on the substrate (or on an acceptor Ub) toward the Ub‐E2 thioester, resulting in the formation of an isopeptide bond [56]. This cascade yields mono‐, multimono‐, or poly‐Ub modifications. Chain topology is determined by conjugation to any of Ub's seven Lys residues (K6, K11, K27, K29, K33, K48, K63) or to the N‐terminus, thereby dictating distinct cellular fates. Polyubiquitinated substrates earmarked for degradation are recognized by the 26S proteasome, whereas deubiquitinating enzymes (DUBs) recycle Ub by hydrolyzing the isopeptide linkage, ensuring Ub homeostasis. Ubiquitination critically governs protein localization, stability, activity, interaction, and degradation and is implicated in diverse cellular processes including cell cycle progression, proliferation, apoptosis, and differentiation [57].

#### 3.1.3 Acetylation/Methylation Modification of Histone Proteins

Eukaryotic DNA is packaged around histone proteins, resulting in tightly packed chromatin. Dynamic modifications of histones govern this packaging to regulate cell fates and functions.

Lys residues within the N‑terminal tails of histones serve as major sites for diverse PTMs. These modifications, including acetylation and methylation, are catalyzed by specific “writer” enzymes: histone acetyltransferases (HATs) and histone methyltransferases (HMTs). Chromatin‐binding proteins known as “readers” specifically recognize these histone tail PTMs and are recruited to distinct genomic loci to regulate genome activity [58]. Conversely, “eraser” enzymes, including histone demethylases (HDMs) and histone deacetylases (HDACs), actively remove these covalent modifications [59]. The addition of the acyl group masks the positive charge on the Lys residue, thereby reducing the affinity of the tail for chromatin, leaving the underlying DNA more exposed. The newly installed acyl group additionally creates a docking surface for bromodomain‐containing transcriptional coactivators. In combination, these mechanisms establish an open chromatin architecture that is permissive to transcription. Histone Lys and arginine residues can also be methylated and can accept up to three methyl moieties, generating mono‐, di‐, or trimethylated species (me1, me2, me3) without altering net charge. The transcriptional consequence, activation or repression, is determined by the genomic locus and the extent of methylation.

**FIGURE 2:**
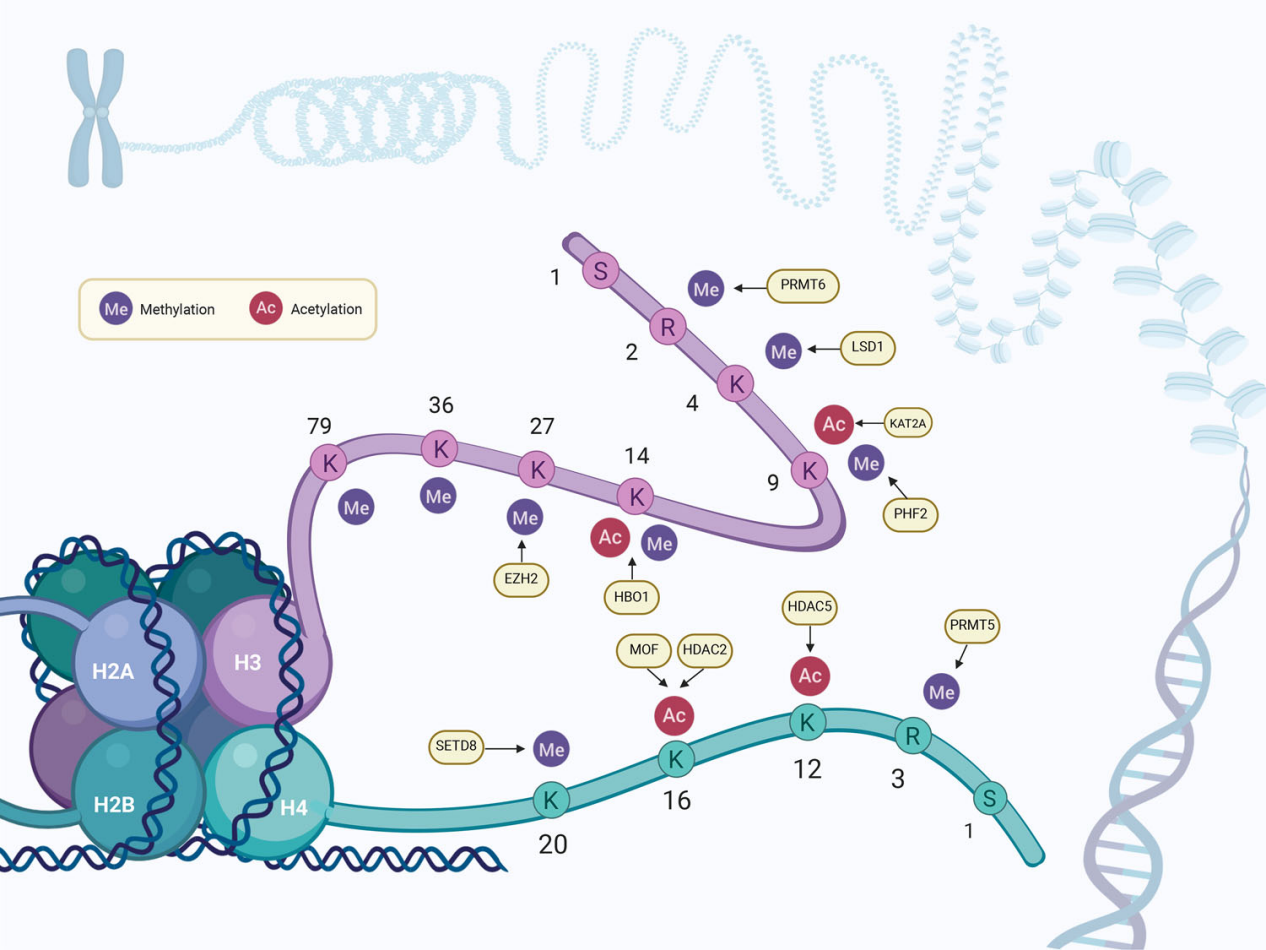
Acetylation and methylation modifications of histone proteins and the enzymes that mediate addition or deletion the chemical groups. Compiling frequently observed posttranslational modification sites on histones H3 and H4 together with their corresponding “writers” and “erasers.” Methylation (Me, red) and acetylation (Ac, purple) are “written” by methyltransferases (e.g., EZH2, SETD8, PRMT5/6) and acetyltransferases (KAT2A, MOF), respectively, and can be “erased” by demethylases (LSD1, PHF2) or deacetylases (HDAC2/5). Dynamic regulation of these modifications governs chromatin architecture and gene expression.

As shown in Figure 2, the acetylation and methylation of histone proteins are linked to their common enzyme transferases/de‐transferases. Activation marks such as histone 3 Lys 4 di‑ and trimethylation (H3K4me2/me3), H3K9 acetylation (H3K9ac), and H4K16ac, as well as the repressive mark H3K27me3, are enriched near transcription start sites and proximal promoter regions [60]. Elongation marks, such as H3K36me3 and H3K79me2, are found predominantly along the bodies of genes and could serve to stop unintended transcriptional initiation within the gene bodies [61, 62, 63].

#### 3.1.4 SUMOylation

SUMOylation is a conserved dynamic protein PTM process. SUMO (small Ub‐like modifier) conjugation is mediated by a multistep enzymatic cascade comprising the SUMO‐activating enzyme (E1) AOS1/UBA2, the sole SUMO‐conjugating enzyme (E2) UBC9, and three families of SUMO E3 ligases, which collectively catalyze the covalent attachment of SUMO to substrate proteins [64]. In contrast to ubiquitination, which primarily targets proteins for proteasomal degradation, SUMOylation regulates protein function without promoting protein breakdown. Instead, it is known to influence protein subcellular localization, stability, and activity, thereby significantly impacting a plethora of cellular processes [65].

#### 3.1.5 Glycosylation

Protein glycosylation, the covalent attachment of glycans to proteins, is an essential PTM that profoundly modulates protein conformation, solubility, stability, and activity. It also plays a role in protecting proteins from proteolysis and directing their subcellular localization [66].

Unlike the linear, template‐directed assembly of proteins or DNA, glycan biosynthesis is nontemplated and combinatorial: monosaccharide units can be linked through varied stereochemistries, anomeric configurations, and glycosidic positions. Mammals elaborate this structural diversity using a minimal alphabet of only 10 monosaccharides—fucose, galactose, glucose (Glc), N‐acetylgalactosamine (GalNAc), N‐acetylglucosamine (GlcNAc), glucuronic acid, iduronic acid, mannose (Man), sialic acid, and xylose—that are assembled along highly branched and tightly regulated biosynthetic routes.

N‐ and O‐linked glycosylation are the most common forms of glycosylated conjugate present in cells. N‐linked glycosylation initiates in the endoplasmic reticulum (ER). A 14‐sugar oligosaccharide (Glc_3_Man_9_GlcNAc_2_) is preassembled on the lipid carrier dolichol‐phosphate and transferred en bloc by the oligosaccharyltransferase complex to an Asn residue within the consensus Asn‐X‐Ser/Thr motif of a nascent polypeptide. After transfer, Glc and Man residues are trimmed in the ER, the glycoprotein undergoes folding surveillance, and then it is trafficked to successive Golgi cisternae where further processing yields high‐Man, hybrid, or complex‐type structures. In contrast, O‐linked glycosylation occurs entirely in the Golgi apparatus. The first sugar, typically GalNAc, is added directly to the hydroxyl oxygen of Ser or Thr by members of the ppGalNAc‐T enzyme family. Subsequent monosaccharides are appended stepwise to generate one of eight core structures (Core 1—8), which can be further elongated, sialylated, fucosylated, or otherwise modified to produce mucin‐type or other O‐glycan classes. Together, these two pathways generate structurally diverse glycans that critically modulate protein folding, stability, trafficking, and intercellular recognition.

### 3.2 Erythroid‐Specific Functional Networks

#### 3.2.1 Signaling Pathways Associated With Phosphorylation Modification in Maintains Normal Differentiation and Maturation of Erythrocytes

Protein phosphorylation is a key regulatory mechanism controlling cellular signaling and is essential for the dynamic behavior and fate determination of erythroid cells. It is through these phosphorylation events that cells respond to erythropoietin (EPO) and other signals, navigate through the cell cycle, and ultimately differentiate into mature erythrocytes. Dysregulation of protein phosphorylation can lead to various erythroid disorders, underscoring its importance in the fine‐tuned control of erythropoiesis. Phosphoproteomic technology provides a set of functionally pertinent phosphosites during erythropoiesis, reflecting the stage‐specific metabolic requirements of erythroid cells, as we briefly describe for the major signaling pathways involved in erythropoiesis below (Figure S2).

Activation of the EPO receptor (EPOR) leads to cell signaling in erythroid progenitor cells. This activation pathway is mediated by the phosphorylated Janus Kinase 2 (JAK2), which then interact with the intracellular regions of the receptor and activate the phosphatidylinositol 3‐kinase (PI3K)/AKT, MAPK, and signal transducer and activator of transcription 5 (STAT5) molecules by a phosphorylation cascade [67]. In Jak2‐deficient mouse embryos, severe anemia is observed, and most embryos die around embryonic Day 12.5, with no enucleated erythrocytes detected. Erythropoiesis is disrupted at the differentiation stage to acidophilic erythroblasts, underscoring the crucial role of JAK2 in this process [68]. Subsequently, phosphorylated by activated JAKs at tyrosine residues, pSTAT5 dimers enter the nucleus to activate numerous biologically important erythroid target genes [69, 70]. These genes and their protein products are predominantly involved in epigenetic regulation, mRNA splicing, signal transduction, EPOR turnover, and negative feedback regulation of STAT5 signaling [71, 72, 73].

The erythroid cytokine receptors c‐Kit and EPOR share considerable overlapping signaling pathways but have distinct roles in erythropoiesis. Tyrosine phosphorylation in the c‐Kit cytoplasmic domain, stimulated by the stem cell factor (SCF), has been shown to promote the proliferation and delay the differentiation of hematopoietic precursor cells. c‐Kit levels decline after the ProE stage, being downregulated relatively early in erythroid maturation compared with EPOR/JAK2 [74]. Research by Haas et al. suggest that the downregulation of SCF/c‐Kit signaling is a prerequisite for EPOR/STAT5‐governed terminal differentiation [75]. Notably, EPO induces phosphorylation of thioredoxin interacting protein (TXNIP) at residues T349 and S358, which elevates c‐Kit expression and suggests a functional crosstalk between TXNIP and c‐Kit. However, the precise regulation of c‐Kit and EPOR expression as well as signaling during erythropoiesis is not fully understood and remains an area of active research.

The MAPK family of kinases is another large and impactful group of signaling molecules activated upon c‐Kit and EPO stimulation [76]. The protein expression in this family varied across erythroid maturation with MAPK1 (ERK2), MAPK3 (ERK1), and RAF1 persisting into late maturation stages. Activating phosphorylation of MAPK1 (ERK2) at T185/Y187 peaks during the Pro‐E/Baso‐E stages and diminishes by the late Baso‐E stage [52]. The lack of any RAS/MAPK signaling components mainly damage the erythroid progenitors and cause a block at the last steps of erythroid cell differentiation [77].

PI3K interacts with the activated EPOR through its SH2 domains. Serine/threonine (Ser/Thr) kinase AKT from the cytosol to the plasma membrane, where it becomes activated. Activated AKT then leading to stimulate its downstream effectors, such as the mechanistic target of rapamycin (mTOR) and its substrates S6 kinase, eukaryotic translation initiation factor 4E‐binding protein 1 (4EBP1) and p70S6K. PI3K/AKT/mTOR mainly protect cells from apoptosis and to mediate EPO‐induced proliferation [78].

Other kinases and phosphatases are also coordinately expressed with distinct kinetics throughout erythrocyte maturation. Proto‐oncogene tyrosine‐protein kinase is an additional kinase involved in EPO signaling cascade by targeting STAT5 activation [79]. Phosphohistidine phosphatase 1 acts as a histidine phosphatase, with EPO‐induced phosphorylation at Y93 site in its phosphatase domain. Protein tyrosine phosphatase nonreceptor type 18 (PTPN18) is highly expressed in hematopoietic progenitors. PTPN18 has been shown to enhance not only STAT5 signaling for EPO‐dependent hematopoietic cell growth but also the activities of the PI3K and MAPK pathways [71]. Oxidative stress responsive kinase 1 is also the most abundant protein kinase, which is indeed active in erythrocytes. It phosphorylates and activates or inhibits the activities of membrane cotransporters and improved membrane deformability [80, 81].

CDKs, a family of Ser/Thr protein kinases, regulate cell cycle progression in erythropoiesis. Inhibition of CDK4/6 leads to cell cycle arrest, prompting cells to exit proliferation and enter differentiation [82]. The cooperative activity of CDK2 and CDK4 extends cell division, facilitating extensive proliferation of hemoglobinized cells [83]. Phosphorylated CDK9, highly expressed in the BFU‐E stage, is a key regulator; its inhibition reduces BFU‐E and CFU‐E proportions and cloning capacity [84]. However, CDK inhibition, such as by the CDK inhibitor p57KIP2, is crucial for maintaining the viability of self‐renewing CFU‐E progenitors in the G0 phase. The balance of CDK activity and inhibition is critical for erythropoiesis, ensuring proper erythroid progenitor proliferation and differentiation into mature erythrocytes.

#### 3.2.2 Signaling Pathways Associated With Ubiquitination Modification in Regulating Erythroid Cell Proliferation and Maturation

The Ub–proteasome system (UPS) is highly active and was first discovered in reticulocytes, where it plays a critical role in eliminating proteins other than globin during reticulocyte maturation [85]. Current research has found that ubiquitination is regulated at different levels by E1, E2, E3, and a series of DUBs and have been implicated in regulating protein stability and turnover in erythroid cell proliferation and maturation [86]. The biological functions of these enzymes in erythropoiesis are summarized in Table S1.

UBE2H as an E2 Ub‐conjugating enzyme is strongly induced during the erythrocyte differentiation of primary human CD34+ cells and is also shown to be upregulated during the differentiation of erythroblasts to reticulocytes, with its expression levels declining during the terminal stages of differentiation [87]. The protein abundance of UBE2H is contingent upon the catalytic activity of its associated C‐terminal to LisH (CTLH) E3 Ub ligase, which is implicated in the enucleation process at the orthochromatic reticulocyte stage [88]. WD Repeat Domain 26 (WDR26) is a CTLH complex subunit that is highly expressed in the hematopoietic tissue of the bone marrow. It regulates the polyubiquitination of a fraction of nuclear proteins, including Lamin B. Studies involving the knockdown of WDR26 have elucidated that an excess accumulation of Lamin B, which is crucial for the initiation of nuclear pores, results in a compromised formation of nuclear openings and hindered nuclear condensation during the erythroblast differentiation process in vivo [89, 90]. As an E2–E3 hybrid enzyme, UBE2O functions autonomously and remains active well into the reticulocyte stage. Its unique structure equips it with multiple substrate recognition domains, allowing it to target a wide range of substrates, including ribosomal proteins and surplus α‐globin chains, for proteasomal degradation. This broad substrate specificity promotes reticulocyte maturation [85, 91]. Trim58, a member of the E3 Ub ligase superfamily, is located within the first intron—a region enriched for erythroid enhancers that bind key erythroid transcription factors such as GATA1 and SCL/TAL1. It was also induced during the late stage of erythropoiesis and ubiquitinates dynein and promotes its proteasomal degradation [92].

E3 Ub ligases, characterized by their RING domain interactions, play pivotal roles in the regulation of globin gene expression and apoptosis during early erythroid development. Representative examples include Trim28 [93, 94], Trim10 [95], and Mdm2–Mdm4 complex [96, 97]. SKP1–CUL1–F‐box protein Ub ligase complex that contains the orphan F‐box protein FBXO11 eliminates transcriptional repressor and then enhances GATA1 occupancy and erythroid gene expression [98]. Liang et al. reported that USP7, a DUB, interacts directly with GATA1 and catalyzes the removal of K48‐linked polyubiquitination chains from GATA1, thereby stabilizing the GATA1 protein. Besides USP7, many other DUBs are also expressed in erythroid cells, albeit at lower levels than USP7 [99]. Future studies are warranted to characterize the functional roles of additional UPS components during erythroid differentiation.

#### 3.2.3 Histone Proteins Acetylation/Methylation Modification Regulates Erythroid Cell Differentiation

Throughout erythropoiesis, both acetylation and methylation modifications of histone proteins are integral to the process, ensuring proper gene expression and cell differentiation.

HBO1 (MYST2/KAT7) is a member of the MYST family of HATs and plays a crucial role in regulating H3K14 acetylation (H3K14ac), which is essential for maintaining stem cell quiescence and self‐renewal. The loss of HBO1 in adult mice can lead to hematopoietic failure due to HSCs exhaustion and the loss of progenitor cells [100]. Additionally, plant homeodomain finger 2 (PHF2), a member of the JmjC family of HDMs, demethylates H3K9me2 and is similarly required for the maintenance of HSC quiescence. PHF2 binds to the p53 promoter, regulating p53 expression by demethylating H3K9me2 in the promoter region, which has been found to be important for HSCs quiescence [101].

SET domain‐containing Lys methyltransferase 8 (SETD8) is the sole methyltransferase in mammals capable of catalyzing the formation of H4K20me1; its expression is upregulated upon erythroid commitment, and disruption of SETD8 impairs erythroid colony‐forming ability [102]. This impairment is associated with upregulated GATA2 expression, which is attributed to a reduction in the H4K20me1 mark on the GATA2 promoter [103, 104]. H3K4me demethylase KDM1A/LSD1 blocks the activity of the myeloid transcription factors and promotes erythroid progenitor differentiation [105]. Other factors that influence erythroid commitment include MOZ/KAT6A (HAT) [106], MOF/KAT8 (H4K16 acetyltransferase) [107], KAT2A (H3K9 acetyltransferase) [108], and DOT1L (HMT) [109]. EZH2, functioning as a histone methyltransferase, catalyzes the modification of H3K27me3 and downregulates the expression of various CDK inhibitors. EZH2‐defunctionalized groups decrease the number and size of BFU‐E and CFU‐E clones and delay differentiation during early erythroid development [110]. PRMT6 is a member of the PRMT family, which consists of enzymes that methylate arginine residues on proteins, including histones [111]. The asymmetric dimethylation of histone H3 at arginine 2 (H3R2me2a) mediated by PRMT6 counteracts the activating H3K4me3. PRMT6 associates with Runt‐related transcription factor 1 on megakaryocytic target genes in progenitor cells and is present on erythroid genes during megakaryocytic differentiation. Herkt et al. found that PRMT6 represses erythroid gene expression during lineage differentiation. Under conditions permissive for erythroid or megakaryocytic differentiation, PRMT6 knockout in vitro enhances erythropoiesis, while PRMT6 overexpression inhibits erythropoiesis in in vitro CFU assays [112]. Another important member of the PRMT family, PRMT5, catalyzes the symmetric dimethylation of arginine residues in histones (at H2A/H4R3 and H3R8) [113]. Conditional knockout of PRMT5 in HSCs from mouse bone marrow disrupts the quiescent state and leads to the exhaustion of HSCs [114].

Histones undergo various modifications during the gradual chromatin condensation and nuclear remodeling in terminal erythropoiesis. SETD8 is also expressed throughout terminal erythroid maturation, promoting mitotic chromatin condensation, with its expression declining at Ortho‐E stage when chromatin condensation is largely completed [115]. Furthermore, in late‐stage mouse fetal erythropoiesis, downregulation of HDAC2 leads to an increase in H4K16ac and demonstrates defects in contractile actin ring formation compared with that of the control. Additionally, HDAC5‐knockdown cells exhibit no defects in these processes [116]. However, Wang et al. suggested that HDAC5 deficiency leads to increased H4K12ac, together with decreased chromatin condensation, supporting a role for HDAC5 in chromatin condensation during erythropoiesis. Nevertheless, the function of HDAC5 in murine erythropoiesis remains controversial [117].

In summary, HATs exert crucial functions during lineage commitment by opening up the chromatin, making it accessible for transcription factors and other regulatory proteins. Conversely, HDACs are instrumental in the terminal phases of erythropoiesis, where they may induce transcriptional silencing in preparation for nuclear condensation [118]. HMTs primarily function during the proliferative stages of erythroid differentiation, contributing to the regulation of gene expression essential for erythroid lineage progression [62].

#### 3.2.4 SUMOylation Process Modulates Protein Properties in Erythropoiesis

SUMOylation of zinc finger protein 521 participates in the regulation of hematopoietic reconstitution in irradiated recipient mice [119]. Erythroid Krüppel‐like factor (EKLF), which plays a critical role in a specific subset of hematopoietic cells, particularly in the erythroid lineage and megakaryocyte‐erythroid progenitors, is covalently modified at Lys 74 by the SUMO‐1/Protein Inhibitor of Activated STAT (PIAS1)/UBC9 pathway. SUMOylation enhances the repressive activity of EKLF, leading to a significant reduction in megakaryocyte numbers and impairment of the bone marrow's capacity to form megakaryocytic colonies [120]. Further studies employed the homozygous KLF1 mouse model to investigate the in vivo role of SUMOylated KLF1 and found that SUMO‐mediated gene repression by EKLF specifically contributes to lineage commitment during hematopoiesis [121]. Another key player in erythroid regulation, GATA1, is also a target of SUMOylation at Lys 137 through the action of GST–SUMO‐1 [122]. SUMO conjugation to GATA1 represses the transcription of GATA1‐inducible genes (EPOR and β‐hemoglobin); consistently, ChIP assays demonstrated impaired recruitment of SUMO–GATA1 to the promoters of EPOR and β‐hemoglobin [123].

Members of the PIAS family serve as an E3 ligase in SUMOylation. However, SUMOylation‐independent negative regulation by PIAS on transcriptional regulators’ target genes during erythropoiesis has also been detected. It is a novel mode of regulation of erythroid lineage gene expression [122, 124]. Future studies integrating genetic approaches, functional analyses, and in vivo models will further advance our comprehensive understanding of the role of SUMOylation machinery in erythropoiesis.

#### 3.2.5 The Dynamic Changes in Glycosylation Level Drive Differentiation and Protein Localization in Erythropoiesis

Mutations in the SEC23 Homolog B (SEC23B) gene, which are inherited in an autosomal recessive pattern, have been identified as a cause of congenital dyserythropoietic anemia (CDAs). These biallelic pathogenic variants lead to impaired glycosylation, culminating in aberrant Golgi apparatus processing in erythroblasts [125]. Structural data of erythrocyte N‐glycans suggest that the loss‐of‐function mutations in SEC23B result in disruptions in N‐glycosylation, which is crucial for the correct localization, activation, and stabilization of proteins in the plasma membrane [126, 127]. Disruptions in this process have been implicated in the dysregulation of the BMP/SMAD signaling pathway, a key regulator of hepcidin transcription. This pathway's impairment is believed to contribute to the pathophysiology of CDAs by affecting iron homeostasis.

The dynamic interplay of O‐GlcNAcylation cycling is essential for the precise modulation of gene transcription. Research has elucidated the fluctuating patterns of O‐GlcNAcylation levels throughout erythropoiesis. Initially, O‐GlcNAcylation levels are low in HSPCs, gradually increasing during the early phases of differentiation and then tapering off as cells approach maturity. Inhibition of O‐linked GlcNAc transferase promotes the differentiation of HSPCs into erythroid progenitor BFU‐E cells. Furthermore, in an effective K562 erythroid differentiation model, which initiates differentiation at the erythroblastic stage, the inhibition of O‐GlcNAcase and the subsequent increase in O‐GlcNAcylation induce erythroid maturation, enucleation, and globin production, particularly β‐globin and, to a lesser extent, α‐globin. BCL11A has emerged as a key mediator of O‐GlcNAcylation‐driven erythroid differentiation and globin production in this context [128, 129]. Significant gaps concerning the roles of O‐GlcNAcylation in early HSCs development and other stages of hematopoiesis warrant further investigation.

## 4 PTMs in Diseases

**FIGURE 3:**
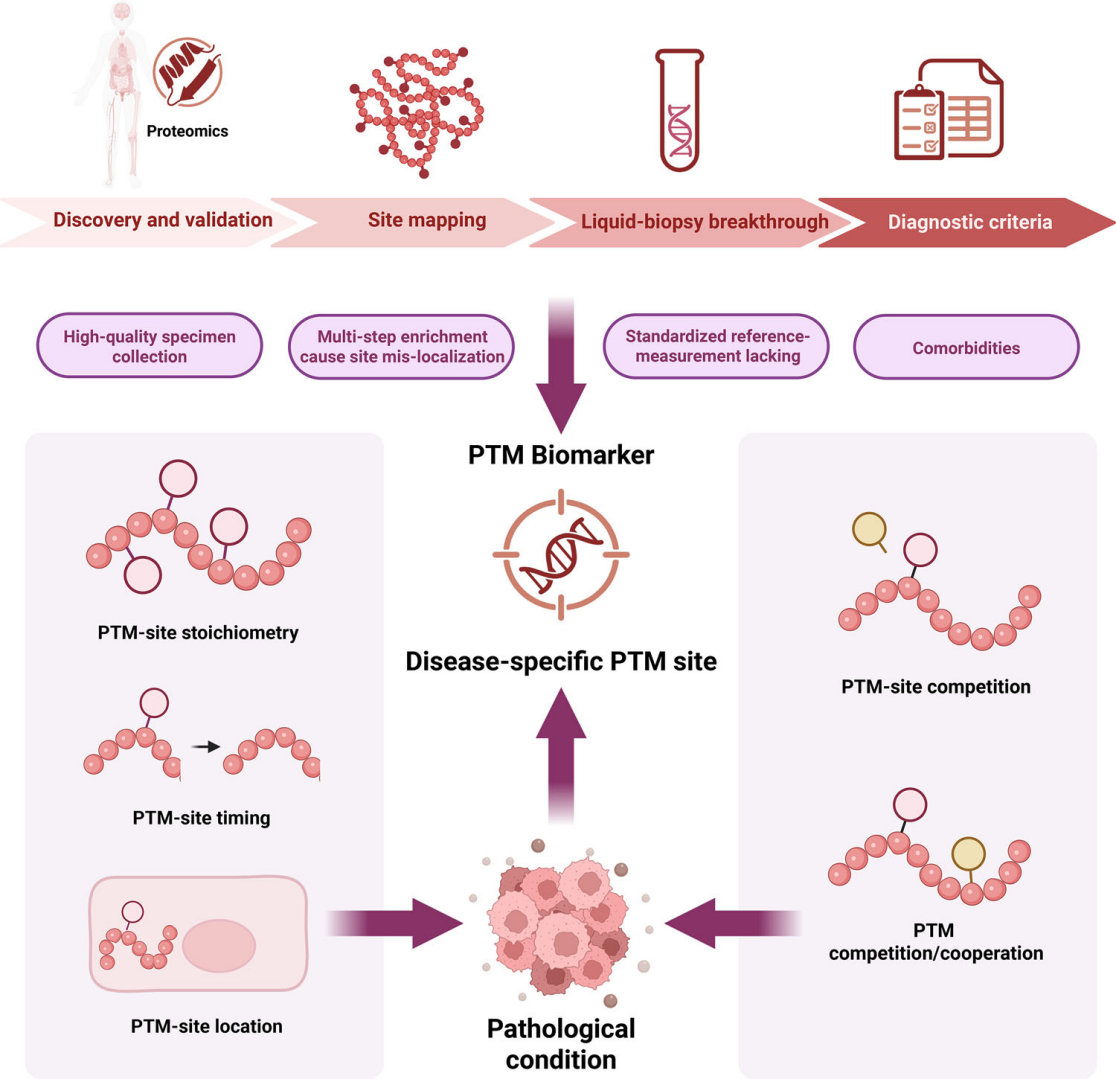
The pathological mechanisms of PTMs and biomarker development. In studies of PTM pathomechanisms, the central challenge is to sift through hundreds to thousands of modification sites and identify the handful that both drive disease phenotypes and are pharmacologically accessible. Turning PTM information on these core sites into clinically useful biomarkers requires a pipeline of discovery, validation of site identity, analytical breakthroughs, and final integration into diagnostic criteria—each step hurdling technical bottlenecks in assay design and sample collection/processing. The contribution of a core‐site PTM to disease progression depends not only on the modification type but also on its spatiotemporal location, stoichiometric level, and the cooperative or competitive crosstalk it engages in with other PTMs.

Given the pivotal physiological roles of protein PTMs, alterations in their abundance or pattern are intimately linked to the onset and progression of numerous diseases. Driven by rapid advances in MS, researchers can now obtain detailed information on relevant PTMs from multiple patient samples [130]. How to distill, from hundreds to thousands of modification sites, the handful that both dictate disease phenotype and remain pharmacologically tractable is the central challenge of PTM‐centric pathological research. Therefore, a comprehensive dissection of PTM sites, their interactome, and the pathological contexts that reshape them is imperative (Figure 3). To translate this principle into mechanistic insight, we utilize erythroid disorders as a clinically tractable paradigm, tracing the precise manner in which PTM events converge on a single transcriptional hub to precipitate the development of ineffective hematopoiesis and defects in erythrocyte function and structure.

### 4.1 General Disease Mechanisms

MS‐based proteomics quantifies global shifts in protein abundance and PTM stoichiometry between healthy and diseased tissues, enabling biomarker discovery and mechanistic dissection of pathological processes. To understand the importance of PTMs in disease, a comprehensive analysis of PTMs in a clinical cohort requires multilayered information, including the type of PTM, the localization of PTMs, the frequency of PTMs in the cohort, and PTM stoichiometry. Functional follow‐up experiments often include in vitro enzyme assays to determine specific enzyme substrates or Lys point mutations to investigate the functional role of a particular PTM [131]. However, current research has moved well beyond merely discovering new PTMs; the central challenge is now to elucidate their functional impact in disease and to pinpoint the most disease‐specific nodes amenable to intervention.

#### 4.1.1 PTM Identification and Biomarker Development in Diseases

As a classic example of a PTM biomarker translated into routine clinical use, the discovery and validation of phospho‐tau (p‐tau) epitomize the archetypal pathway from pathological observation, through site mapping and liquid biopsy breakthroughs, to final incorporation into diagnostic criteria. Three back‐to‐back studies demonstrated that plasma p‐tau217 detects Aβ‐PET positivity with >94 % accuracy, opening the first realistic window for replacing lumbar puncture with a blood test [132, 133]. In 2023, the NIA‐AA revised framework elevated “elevated plasma p‐tau217 plus reduced Aβ42/40 ratio” to the highest level of biological evidence for AD, equivalent to CSF biomarkers [134], and by 2025, a commercial plasma p‐tau217 assay had entered routine laboratory menus. Moreover, dynamic changes in p‐tau217 mirror real‐time target engagement by anti‐tau monoclonal antibodies such as semorinemab, making it the first companion diagnostic used to confirm dosing in tau‐immunotherapy trials [135].

Beyond AD, proteomic approaches have already uncovered a wealth of potential biomarkers and therapeutic targets across a broad spectrum of disorders. In septic cases, enhanced acetylation of p53 is observed in renal tubular epithelial cells; this coincides with a transient elevation followed by a rapid decline in the level of autophagy, aggravating sepsis‐induced acute kidney injury (AKI) [136]. The study has found that metformin could reverse specific histone modification markers, such as changes in histone H3K36me2 [137]. Zhang et al. developed a ML‐based PTM Learning Signature from multicohort analysis of 1231 lung adenocarcinoma cases, identifying beta‐1,4‐galactosyltransferase 2 (B4GALT2) as a key prognostic biomarker within this framework, where elevated B4GALT2 expression correlates with poor survival and CD8+ T‐cell exclusion, suggesting an immunoevasive role in lung adenocarcinoma progression [138]. Collectively, these data underscore the panoramic utility of PTM‐driven biomarkers across pathologies. Continuously integrate multilayer PTM profiling, from proteomics to epigenomics, into large‐scale clinical trials to refine disease subtyping, monitor therapeutic efficacy, and guide precision treatment strategies.

Although a growing number of studies have explored PTM‐based biomarkers, a high hurdle remains between bench‐side discovery and bedside implementation [139]. First, analytical bottlenecks: the low stoichiometry and transient nature of most PTMs demand multistep enrichment, increasing both technical variance and the risk of site mis‐localization. Second, high‐quality specimen collection is critical—postexcision handling can trigger ex vivo modification drift that generates artifactual sites, whereas comorbidities such as aging, diabetes, or concomitant drugs can alter glycosylation or oxidation patterns. Discrepancies also arise from different biopsy sites and sampling protocols. Most importantly, standardized reference measurement procedures are still lacking, preventing the cross‐laboratory verification of data and slowing the regulatory acceptance of these biomarkers [140, 141]. Therefore, whether a PTM‐based biomarker can serve as a clinically applicable tool still faces multiple challenges and requires rigorous validation.

#### 4.1.2 Selective Rewiring of the Same PTM Class Across Distinct Pathological Contexts

During the onset and progression of disease, identical PTM events can lead to distinct pathological outcomes depending on differences in their dose, timing, and subcellular localization.

The role of tau phosphorylation in the nervous system has been extensively characterized. In neurodegenerative diseases, aberrant tau phosphorylation not only causes a loss of function (e.g., reduced microtubule binding) but may also confer toxic gain‐of‐function (e.g., increased tau–tau self‐interaction). Although tau is predominantly expressed in the brain, recent studies have revealed that hyperglycemia drives tau phosphorylation in the pancreas, enhancing the microtubule disassembly and acutely potentiating Glc‐stimulated insulin secretion [142, 143]. This necessitates that p‐tau‐targeting strategies take extra‐neuronal responses into account [144]. Quantitative proteomics has brought PTM‐site stoichiometry to the forefront, yet not every modification is functionally relevant in a given pathological context. For tau, an increased occupancy of specific sites mirrors both the progression and the hallmark pathology of AD [145].

The subcellular location where a modification occurs also dictates the course of pathology. Wang et al. identified monoubiquitylation as the posttranslational switch that ignites the NLRP6 inflammasome: the E3 ligase UBE2O attaches single Ub moieties to two clusters—K680‐687 and K115‐130—on NLRP6. Critically, monoubiquitylation at K115‐130 exerts its effect only when this segment resides in the cytosol; there, the Ub tail creates a steric block that prevents importin‐α1 binding. If mutations or a strong nuclear‐localization signal relocate K115‐130 into the nucleus, the same monoubiquitylation event fails to disrupt the NLRP6–importin‐α1 interaction, and inflammasome assembly is aborted [146].

Collectively, these findings underscore that the pathological impact of any PTM is not dictated by its chemical identity alone. Instead, a three‐dimensional code—stoichiometry (how much), timing (when), and geography (where)—determines whether the same PTM will protect, perturb, or even switch physiological signaling to disease‐driving cascades.

#### 4.1.3 Functional Integration of Multimodification Crosstalk Within the Same Pathological Context

Disease progression is not governed by a single PTM; instead, different modifications engage in positive cooperation or negative competition, and their integrated crosstalk ultimately determines cell fate and the direction of pathology.

O‐GlcNAcylation and phosphorylation can occur at Ser or Thr residues; it is crucial that the interplay between these two modifications is vital to bioenergetic and biosynthetic demand. Crosstalk between O‐GlcNAcylation/phosphorylation comes in many flavors, for instance, by competition for the same site/residue (reciprocal crosstalk), as well as by modifications influencing each other in proximity or even distal on the protein sequence. PTM crosstalk is also observed on the writers of these modifications (i.e., kinases and O‐GlcNAc transferase), on the erasers (i.e., phosphatases and O‐GlcNAcase), and on the readers and the substrates. Zhou et al. have shown that the Glc‐sensitive kinase unc‐51‐like autophagy activating kinase 1 (ULK1) binds PKM2 and phosphorylates it at Ser333. This phosphorylation blocks PKM2 O‐GlcNAcylation, stabilizes the active tetramer, enhances pyruvate‐kinase activity, curtails nuclear import, and thereby tunes the Warburg effect in breast‐cancer cells [147]. In diabetic retinopathy, robustly elevated O‐GlcNAcylation in retinal endothelial cells suppresses phosphorylation of the Hippo effector Yes‐associated protein (YAP) at Ser397. By blocking this inhibitory mark, O‐GlcNAcylation locks YAP in its active state, driving a transcriptional program that promotes angiogenesis and Glc metabolism and ultimately fuels retinal vascular dysfunction [148]. Similar examples are found in multiple PTM crosstalk events, such as the cooperative promotion of prostate cancer progression by ubiquitination and phosphorylation [149] and the control of phosphoserine aminotransferase 1 protein stability in lung adenocarcinoma through the interplay between acetylation and ubiquitination [150]. In disease research, it is imperative to recognize the complexity of PTMs crosstalk; identifying the critical PTM‐combinations offers fresh molecular targets and intervention strategies for precision medicine and individualized therapy.

### 4.2 Erythroid Pathologies

**FIGURE 4:**
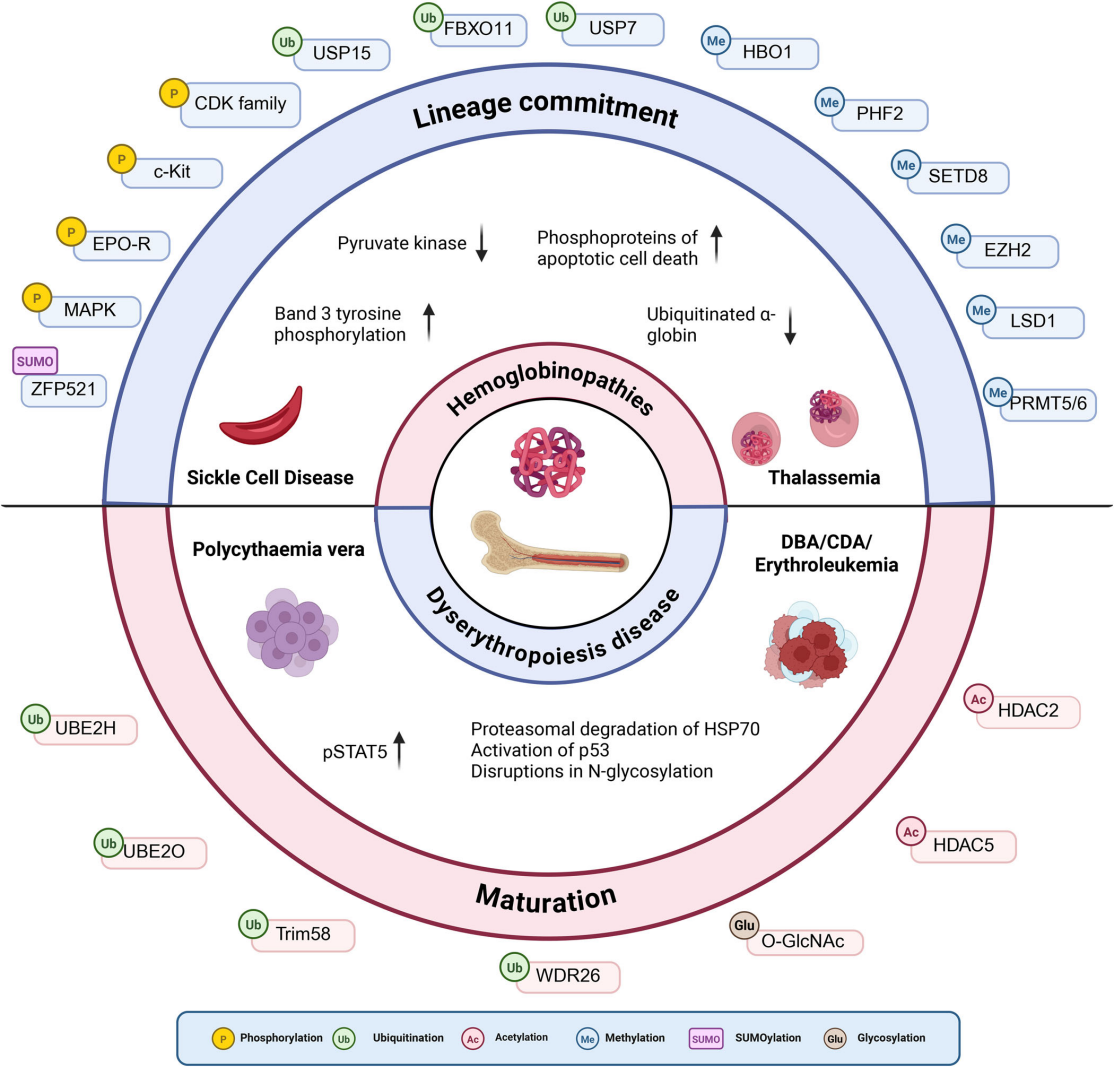
Types of PTMs and their targets involved in the various stages of erythropoiesis with the pathological mechanisms involved in erythroid diseases. The major PTM targets and types involved in erythroid development that have been identified in recent studies. Mutations in genes within PTM network can lead to aberrant erythropoiesis and deficiency erythrocyte output.

Given the intricate and highly regulated nature of erythropoiesis, erythroid disorders can generally be classified into two categories: those with the most significant defects occurring during the differentiation process, such as diseases characterized by ineffective erythropoiesis, and those in which differentiation proceeds more or less normally but with defects in the function or structure of the mature RBCs. The dysregulation of protein modifiers, often stemming from genetic mutations in the corresponding genes, can have profound clinical implications. Such mutations may occur either in the germline or somatically in HSCs or during distinct developmental stages. Typically, the most pronounced phenotypic effects and clinical symptoms become evident after commitment to the erythroid lineage. Aberrant modifications of proteins are linked to conditions such as anemia, erythroid dysplasia, and clonal hematopoiesis [62] (Figure 4).

#### 4.2.1 Protein Modifications in Hemoglobinopathies

A quintessential example is that loss of UBE2O expression, as observed in a β‐thalassemia mouse model, leads to reduced levels of ubiquitinated α‐globin, thereby alleviating the toxic effects of unbalanced α‐globin accumulation and extramedullary erythropoiesis [151]. These definitive results demonstrate the biological and clinical relevance of the PTM activity of UBE2O and suggest that the UBE2O–Ub axis constitutes a potential therapeutic target. Phosphoproteomic studies of bone marrow HSCs/CD34+ cells derived from individuals with HbE/β‐thalassemia have identified an elevated abundance of phosphoproteins typically associated with apoptotic cell death. Notable among these are cytochrome *C*, caspase 6, and apoptosis‐inducing factors, which are integral to both mitochondrial‐dependent and death receptor‐mediated apoptotic pathways [152].

Comparative studies in sickle cell disease (SCD) patients before and after hydroxyurea (HU) treatment, together with analyses comparing SCD patients and healthy controls, demonstrate widespread protein phosphorylation and ubiquitination in SCD, and that HU treatment attenuates these modifications. Several of these phosphorylated proteins are important for microparticle formation, which is a hallmark of SCD pathology, including Band 3, adducin, ankyrin 1, spectrin, and Band4.1 [153, 154]. The tyrosine phosphorylation of Band 3 promotes erythrocyte membrane weakening that causes release of both membrane vesicles and cell free hemoglobin that in turn initiates vaso‐occlusive events. Given this mechanism, the exploration of inhibitors targeting the tyrosine kinases responsible for Band 3 tyrosine phosphorylation represents a potential therapeutic strategy for SCD [155]. Pyruvate kinase (PK) is a key glycolytic enzyme responsible for ATP production. Enhancing PK activity can reduce 2,3‐diphosphoglycerate (2,3‐DPG) levels, increase ATP levels [156, 157]. A clinical study from the Netherlands demonstrated decreased PK thermostability and reduced relative PK activity (PK/HK ratio) in patients with SCD. An elevated 2,3‐DPG content was observed in sickle RBCs compared with healthy controls [158]. This increase promotes HbS polymerization under deoxygenation and thereby induces sickling. The emergence of an activator of erythrocyte PK, through the Rapoport–Luebering shunt‐a unique RBC‐specific glycolytic bypass‐decreases 2,3‐DPG levels and increases ATP production, thereby enhancing hemoglobin‐oxygen affinity and improving the function of sickle RBCs [159].

#### 4.2.2 Protein Modifications in Other Erythroid Diseases

Research into polycythemia vera (PV) pathogenesis has highlighted the significance of aberrant protein phosphorylation signaling. The JAK2 V617F mutation is a key player, occurring in over 90% of PV cases and in about half of those with essential thrombocythemia or primary myelofibrosis [160]. This mutation leads to the hyperactivation of STAT5, disrupting the normal developmental trajectory of hematopoietic stem and progenitor cells and promoting the abnormal proliferation of erythroid progenitors [161]. Advanced analyses, including transcriptomic and phospho‐proteomic studies on erythroblasts from normal and PV patients, have uncovered elevated phosphorylation of kinases in the JAK/STAT, PI3K, and GATA1 pathways, potentially disrupting typical erythroid cell expansion and maturation in PV [162]. Proteasomal degradation of HSP70, the chaperone of GATA1, and the activation of p53 are considered pivotal in Diamond–Blackfan anemia (DBA) pathophysiology and modulators of DBA phenotypes. These mechanisms contribute to the characteristic defects in erythroid proliferation, delayed differentiation, increased apoptosis, and diminished globin expression observed in DBA [163, 164], including the CDAs, as previously discussed. Dysregulation of PTMs constitutes a core mechanism underlying erythroid disorders.

## 5 Therapeutic Targeting of PTMs

Significant strides have been made in understanding the biological and molecular mechanisms of PTMs. This deeper comprehension, integrated with novel chemical insights, has not only established the clinical viability of targeted protein modification but has also spurred substantial growth in therapeutic development. Currently, numerous investigational drugs are in active clinical trials, reflecting the potential of PTM‐targeted therapies. The current clinical or preclinical development of PTM‐targeted drugs can be divided into two main categories: one that inhibits or activates the enzymes responsible for PTMs, and another that directly recognizes or interferes with the modification state. Below, we summarize approved or clinically advanced investigational drugs within each category and describe their applications in erythroid disorders, classified by PTM type.

### 5.1 Broad‐Spectrum Approach

#### 5.1.1 PTM Enzymes Targeting

Remarkable success has been achieved in the development of drugs targeting protein kinases, methyltransferases, HDACs, and Ub ligases.

Eighty‐five small‐molecule protein kinase inhibitors have reached the market until 2025. Of these drugs, five target dual specificity protein kinases (MEK1/2), 14 inhibit protein‐Ser/Thr protein kinases, 21 block nonreceptor protein‐tyrosine kinases, and 45 target receptor protein‐tyrosine kinases (Table 1). Approximately 70% of these agents are indicated for the treatment of malignancies, whereas the remainder are increasingly being investigated for non‑neoplastic disorders, including autoimmune and neurodegenerative diseases [165]. The vast majority of United States Food and Drug Administration (US FDA)‐approved kinase inhibitors exert their effect by occupying the ATP‐binding pocket of the target kinase. The comparatively small number of non‐ATP‐competitive agents reflects the intrinsic difficulty of discovering and developing such compounds. Regardless of mechanism, however, most marketed inhibitors possess limited selectivity and act against multiple kinases. Although ATP‐competitive molecules continue to reach approval, they rarely eradicate the disease; patients inevitably face relapse driven by acquired or innate resistance. Resistance can be pre‐existing or emerge during treatment, and its causes are multifaceted—ranging from point mutations within the kinase domain to activation of bypass signaling pathways and histologic transformation. Among these, single‐amino‐acid substitutions in the kinase domain are the most frequent and fastest mechanism of acquired resistance, often detectable within months to a few years of therapy. Beyond mutations, a major mutation‐independent route to resistance is reactivation of signaling cascades downstream of the inhibited kinase; for example, in chronic myeloid leukemia (CML), reinforcement of PI3K, MAPK, SRC, or JAK/STAT signaling can override BCR–ABL blockade, reinstitute aberrant proliferation, and precipitate disease recurrence [166].

**TABLE 1:** US FDA approved protein kinase inhibitor drug and their targets.

Kinase family	Class of kinase	US FDA approved
ALK	RY	Alectinib, brigatinib, ceritinib, crizotinib, ensartinib, lorlatinib
BCR–ABL	NRY	Asciminib, bosutinib, dasatinib, imatinib, nilotinib, ponatinib
BTK	NRY	Acalabrutinib, ibrutinib, pirtobrutinib, zanubrutinib
B‐RAF	S/T	Dabrafenib, encorafenib, tovorafenib, vemurafenib
CDK4/6	S/T	Abemaciclib, palbociclib, ribociclib, trilaciclib
CSF1	RY	Pexidartinib
EGFR/ErbB	RY	Afatinib, capivasertib, dacomitinib, erlotinib, gefitinib, lapatinib, lazertinib, mobocertinib, neratinib, osimertinib, tucatinib
FGFR	RY	Erdafitinib, futibatinib, infigratinib, nintedanib, pemigatinib
FLT3	RY	Gilteritinib, midostaurin, quizartinib
FKBP	S/T	Everolimus, sirolimus, temsirolimus
JAK	NRY	Abrocitinib, baricitinib, deuruxolitinib, fedratinib, momelotinib, pacritinib, ritlecitinib, ruxolitinib, tofacitinib, upadacitinib
MEK1/2	Y/T	Binimetinib, cobimetinib, mirdametinib, selumetinib, trametinib
MET	RY	Capmatinib, tepotinib
PDGFR	RY	Avapritinib, ripretinib
RET	RY	Pralsetinib, selpercatinib, vandetanib
ROCK	S/T	Belumosudil, netarsudil
ROS1	RY	Repotrectinib
SYK	RY	Fostamatinib
TRKA	RY	Entrectinib, larotrectinib
TYK2	NRY	Deucravacitinib
VEGFR	RY	Axitinib, cabozantinib, fruquintinib, lenvatinib, pazopanib, regorafenib, sorafenib, sunitinib, tivozanib

*Abbreviations*: NYR, nonreceptor protein‐tyrosine kinase; RY, receptor protein‐tyrosine kinase; S/T, protein‐serine/threonine kinase; Y/T, dual specificity protein kinase‐tyrosine phosphorylation followed by threonine phosphorylation of target kinase activation segments.

*Data source*: Protein Kinase Inhibitors | BRIMR.

By irreversibly locking onto poorly conserved, noncatalytic nucleophiles, covalent ligands can parse the kinome with a resolution that ATP‐mimetic reversible inhibitors seldom reach. Exploiting this principle, targeted covalent kinase inhibitors have evolved from a niche tactic into a central pillar of modern drug discovery, simultaneously widening the druggable target space, circumventing gate‐keeper resistance, and shattering the selectivity ceiling imposed by the conserved ATP cleft. The clinical translation of this strategy is already tangible: 11 small‐molecule covalent kinase blockers have secured US FDA approval, spanning acalabrutinib and zanubrutinib (BTK), ibrutinib (BTK), afatinib, dacomitinib, lazertinib, mobocertinib, and osimertinib (mutant EGFR), neratinib (HER2), ritlecitinib (JAK3), and futibatinib (FGFR2) [167, 168]. Different MS‐based proteomics approaches, namely, phosphoproteomics, kinobeads, and thermal proteome profiling, are ongoing efforts directed to decipher the molecular mechanisms of kinase inhibition to improve drug design [169]. Osimertinib is the standard first‐line treatment for EGFR‐mutant non‐small‐cell lung cancer, yet most patients eventually develop acquired resistance that precludes a cure. Phosphoproteomic mapping of drug‐tolerant persister cells shows that resistance is driven by reactivation of EGFR‐downstream signaling and by antiapoptotic rewiring—highlighted by hyperphosphorylation of YAP1 and the mTOR–BAD axis [170]. Casado et al. mined a primary AML phosphoproteome atlas and paired it with global proteomics to identify PF‐3758309 as the most potent PAK inhibitor for curbing proliferation and driving apoptosis in AML cell lines, pinpointing PHF2 pSer705 as a robust pharmacodynamic biomarker of response [171].

Considering the role of HDACs in cancer and diseases, growing interest has been observed over the past years in the pharmaceutical field regarding the discovery of HDAC inhibitors (HDACi). To date, several HDACi have entered clinical trials, while five of them have been approved by the US FDA as drugs to be employed for cancer treatments (vorinostat, panobinostat, romidepsin, and belinostat) and for Duchenne therapy (givinostat) [172]. HDACi development has primarily focused on well‐established pharmacophore models, mainly using hydroxamic acid and 2‐aminoanilide as zinc‐binding groups [173]. Clinically viable therapies targeting the activities of histone Lys methyltransferases (HKMTs) and demethylases (HKDMs) have only recently begun to emerge following the US FDA approval of the EZH2 inhibitor Tazemetostat in 2020 [174], for the treatment of certain patients with epithelioid sarcoma or follicular lymphoma. However, despite the efficacy achieved, many of these compounds have shown metabolic liabilities contributing to toxicity in in vivo models.

The clinical success of cancer treatments targeting the UPS has demonstrated that the UPS is a viable therapeutic strategy. Proteasome inhibitor (PI)‐induced tumor suppression is a classical strategy for cancer treatment. Bortezomib, a prototype PI drug, is effective against malignancies with high UPS dependence, such as multiple myeloma (MM) and mantle cell lymphoma [175]. Marizomib is a more lipophilic and less neurotoxic drug. In a Phase 3 trial, marizomib is being assessed for the treatment of malignant glioblastoma in combination with temozolomide and radiotherapy [176]. The main challenges associated with PI drug are acquired resistance and low efficacy due to poor pharmacokinetic/pharmacodynamic profiles. In addition, pharmacological inhibition of E1‐activating enzymes may produce nonspecific effects, but they can be exploited for short‐term treatment within a well‐defined safety window. By contrast, E3 ligases and/or DUBs have long been the main focus of research because of their inherent substrate selectivity. Nevertheless, features of E3s—such as the absence of a canonical catalytic site, extensive PPIs, and multidomain architectures—have hindered the development of potent inhibitors. Only a handful of DUB inhibitors, exemplified by VLX1570, have entered clinical trials (Phase 1), yet these were prematurely discontinued because of severe toxicity [177]. Mitoxantrone, an US FDA‐approved agent, inhibits the DUB Ub‐specific peptidase 11 (clinical trial identifiers: NCT02724163, NCT05313958), while putative inhibitors of DUB‐specific peptidase 14—namely 6‐mercaptopurine (NCT00866918, NCT00482833) and 6‐thioguanine (NCT05276284, NCT00549848)—are currently under clinical investigation for various malignancies, including leukemia [178].

#### 5.1.2 PTM State Targeting

However, nontargeted perturbation activities may introduce unwanted off‐target toxicity, thereby limiting the clinical utility of these drugs. The development of PTM drugs targeting more precise sites has become a top priority. Meng et al. introduced “Post‐translational Modification Inspired Drug Design (PTMI‐DD)” as a strategy to bridge chemical and biological spaces by targeting PTM protein isoforms, including covalent inhibitors mimicking PTMs and targeting distinctive binding sites [179]. Zhang et al. demonstrated this approach in kinases by developing ML models to predict PTMs, identifying their enrichment in allosteric pockets, and successfully creating the covalent inhibitor DC‐Srci‐6668 that targets the PTM pocket of c‐Src kinase [180]. Chemically induced proximity (CIP) has recently emerged as a powerful research tool and been used to target a single PTM event on the target protein. CIP technologies such as proteolysis targeting chimeras (PROTACs), molecular glue degraders, LYTACs, and autophagy‐targeting chimeras promote targeted protein degradation (TPD) by poly‐ubiquitinating the protein of interest (POI) or recruiting it directly to the 26S proteasome or lysosome [181]. Collectively, these advances mark a paradigm shift from simply “drugging the enzyme” to “drugging the modification state” positioning PTMs themselves as the next dimension of drug selectivity. Continued integration of chemoproteomic surveillance, AI‐guided pocket prediction, and proximity‐based pharmacology will be essential to convert the remaining PTM code into a druggable space, ultimately expanding the therapeutic index and delivering precision medicines that discriminate between healthy and pathologically modified proteoforms [182].

### 5.2 Erythroid‐Specific Interventions

These novel insights into PTMs also provide the theoretical foundation for the development of new treatments for erythroid diseases. Currently, the most extensively studied PTM‐target drugs in erythroid disorders mainly involve phosphorylation modifications (Table 2), focusing primarily on two categories: JAK inhibitors and pyruvate activators. Ruxolitinib, a selective JAK inhibitor, is currently used as a second‐line cytoreductive treatment for PV; clinical trials have shown that it is a safe and effective long‐term treatment option, especially for HU‐intolerant/refractory PV (HC‐INT/RES PV) [183, 184, 185]. A 5‐year result from a randomized, open‐label, Phase 3 study (RESPONSE) showed that the 10 mg twice per day of ruxolitinib was superior to best available therapy (BAT), including HU, interferon, or pegylated interferon, in achieving hematocrit (Hct) control, splenic response, complete hematological remission, and overall clinical response. Moreover, the benefits of ruxolitinib were not limited to HC‐INT/RES PV patients who initiated ruxolitinib at the beginning. Patients who switched to ruxolitinib after 32 weeks of BAT also observed clinical improvement [184]. Infections were more common in ruxolitinib‐treated patients, particularly respiratory, genitourinary, and cutaneous herpes zoster, due to its immunomodulatory and immunosuppressive effects [186, 187]. Nonmelanoma skin cancers also occurred in the clinical trials, especially in non‐JAK2‐mutated patients exposed to ruxolitinib [188]. Other JAK inhibitors, such as momelotinib, have demonstrated reductions in JAK2 V617F allele burden and disease symptoms in in vitro studies, but their efficacy in clinical trials has been limited [189]. For gandotinib, the overall response rate in patients with JAK2 V617F‐mutated PV was 95%, and it did not exhibit the hematological or infectious toxicities reported with ruxolitinib, nor the neurological toxicities or severe safety concerns observed with other JAK inhibitors [190]. A Phase 2 clinical trial is currently underway for PV patients who have demonstrated intolerance to, failure of primary response to, or disease progression while receiving ruxolitinib (NCT01594723).

**TABLE 2:** Various modifiers developed in recent years for erythroid diseases.

Drug	PTM class	Mechanism	Indication	Study level	Results and comments	References
Imatinib	Phosphorylation	Band 3 tyrosine phosphorylation impairment reduces pain crises	SCD	Clinical trial (NCT03997903)	Study halted due to low accrual; no hematological changes noted	[197, 198]
Ruxolitinib		JAK2 kinases inhibitors	PV	Clinical trial (NCT05853445, NCT05421104)	Treatment regimen is notably manageable and safe; US FDA approval	[217, 218]
Momelotinib		JAK1 and JAK2 inhibitor	PV	Clinical trial (NCT01236638 NCT01998828)	Terminated due to limited efficacy	[189]
Lestaurtinib		JAK2 kinases inhibitors	PV	Clinical trial (NCT00586651)	Poorly tolerated; primary endpoint not met	[219]
Gandotinib		JAK2 kinases inhibitors	PV	Clinical trial (NCT01134120, NCT01594723)	Demonstrating efficacy in JAK2 V617F‐mutated MPNs	[190]
Mitapivat		Pyruvate kinase activator	SCD	Clinical trial (NCT05031780)	Well‐tolerated and effective	[193]
			Erythrocyte membranopathies and CDA II	Clinical trial (NCT05935202)	Preliminary improvements in hemoglobin levels and markers of hemolysis	[220]
			Thalassemia	Clinical trial (NCT04770779)	High hemoglobin response rate and good tolerability	[221]
Etavopivat		Pyruvate kinase activator	SCD	Clinical trial (NCT03815695)	High hemoglobin response rate and good tolerability	[195]
Tebapivat		Pyruvate kinase activator	SCD	Clinical trial (NCT04536792)	Deemed safe in healthy volunteers and SCD patient	[194, 222]
RN‐1	Methylation	LSD 1 inhibitor	SCD	Animal	Normal baboons showed increased HbF with prolonged treatment tolerance.	[205, 223]
			Thalassemia	Cell	Increased in γ‐globin transcript and HbF expression	[224]
ORY‐3001		LSD 1 inhibitor	SCD	Animal	Increased in γ‐globin transcript and HbF expression	[203]
OG‐S1335		LSD 1 inhibitor	SCD	Animal	Increased in γ‐globin transcript and HbF expression	[204]
INCB059872		LSD 1 inhibitor	SCD	Clinical trial (NCT03132324)	Terminated due to business decision	
UNC0638		EHMT1/2 inhibitor	Thalassemia	Cell	Inducing HbF expression	[206]
DZNep		S‐adenosylmethionine‐dependent methyltransferase inhibitor	Hematological diseases	Cell	Promoting erythroid differentiation	[209]
IOX1		EHMT inhibitor	Thalassemia	Cell	Selective silencing of α‐globin	[207]
A‐366		Inhibitor of the G9a methyltransferase and the chromatin reader Spindlin1	Erythroleukemia	Cell	Favoring erythroid differentiation	[225]
Vorinostat	Acetylation	HDAC inhibitors	SCD	Clinical trial (NCT01000155)	Terminated due to slow accrual	[226]
			Thalassemia	Cell	Suppressing α‐globin and inducting γ‐globin	[210]
Panobinostat		HDAC inhibitors	SCD	Clinical trial (NCT01245179)	Recruitment	[226]
Sodium valproate		HDAC inhibitors	β‐hemoglobinopathies	Cell	Overexpressing γ‐globin and AHSP	[227]
CT‐101		HDAC inhibitors	SCD	Cell	Inducing HbF expression	[228]
Givinostat		HDAC inhibitors	PV	Clinical trial (NCT06093672)	Well‐tolerated and effective	[213]

*Abbreviations*: MPNs, The Philadelphia chromosome‐negative myeloproliferative neoplasms; LSD 1, lysine demethylase 1; AHSP, alpha‐hemoglobin stabilizing protein; SCD, sickle cell disease; PV, polycythemia vera; CDA, congenital dyserythropoietic anemias.

PK activators, which target the tetrameric R form of RBC PK (PKR) to reduce 2,3‐DPG and increase ATP levels, thereby enhancing hemoglobin‐oxygen affinity and reducing sickling and hemolysis, are emerging as promising therapies for SCD and thalassemia [191]. Currently, several PK activators including mitapivat, etavopivat, and tebapivat are under clinical investigation. In the Phase 3 ENERGIZE‐T study (NCT04770779), a total of 258 patients with transfusion‐dependent α‐ or β‐thalassemia were enrolled, with 171 patients randomized to receive mitapivat 100 mg twice daily and 87 patients randomized to receive a matching placebo. Compared with placebo, mitapivat significantly reduced transfusion burden and demonstrated durable reductions lasting up to 36 weeks (Week 13 to Week 48). Overall results were not driven by any of the individual prespecified subgroups, including genotype and BL transfusion burden [192]. Multiple Phase 2/3 clinical trials of mitapivat are currently planned or ongoing for SCD, erythrocyte membranopathies, and CDA II: NCT05031780, NCT04610866, NCT05675436, NCT06286046, NCT05935202. From the currently published trial results, mitapivat has shown significant potential as a therapeutic option for various hemolytic anemias, with promising efficacy and safety profiles [193]. Other agents, such as etavopivat and tebapivat, have also demonstrated promising efficacy and safety in ongoing Phase 1 or Phase 2 clinical trials [194, 195].

Syk tyrosine kinase inhibitors also warrant consideration as potential treatments for SCD, impairing constitutive tyrosine phosphorylation of Band 3 [196]. Imatinib, an oral tyrosine kinase inhibitor developed for the treatment of chronic myelogenous leukemia, significantly increased Hct and hemoglobin levels and reduced acute and chronic multiorgan damage in humanized sickle cell mice [197]. However, a clinical trial from Iran evaluated the hematological parameters of seven SCD patients before and after 1 year of imatinib treatment, and no changes in RBC and Hb levels were observed [198]. Although the clinical results of imatinib were not ideal, this provided a new perspective on the mechanism by which Band 3 phosphorylation contributes to RBC membrane instability. In another study, based on a Phase 1 trial (NCT04000165), the levels of phosphorylated Band 3 in RBC ghosts were measured in patients treated with different doses of mitapivat. As mitapivat doses increased, phosphorylated Band 3 levels decreased, which was associated with improved RBC membrane integrity and enhanced RBC survival [199]. The extended findings of this study (NCT04610866) also found that mitapivat therapy significantly reduced RBC Band 3 phosphorylation within 2 weeks of exposure and continued to improve over 2 years of treatment, demonstrating that the improvement in RBC deformability in SCD patients induced by Mitapivat is rapid and sustained [200]. Drugs targeting this mechanism still need to be developed and tested.

Most histone methylation and acetylation drugs used in erythroid disorders are in the preclinical research stage (Table 2). LSD1 inhibitors, which are HDMs, are potent activators of fetal hemoglobin (HbF) and among the most promising agents in development to increase HbF levels for the treatment of SCD. Treatment with the LSD1 inhibitors RN‐1, ORY‐3001, and OG‐S1335 could all increase HbF levels and was well tolerated in both normal baboons and SCD mouse models over an extended treatment period [201, 202, 203, 204]. Although INCB059872, also an LSD1 inhibitor, has entered clinical trials for acute myeloid leukemia and small cell lung cancer, it was regrettable that its clinical study in SCD was terminated due to business decisions (NCT03132324). Moreover, increased doses of INCB059872 produce neutropenia and thrombocytopenia, as LSD1 activity is required during terminal hematopoietic differentiation. Therefore, the best strategy may be development of combinatorial drug regimens [205]. In addition to LSD1 inhibitors, other histone methylation modification‐related agents, such as UNC0638, DZNep, and IOX1, have demonstrated promising potential in the cellular assay stage [206, 207, 208, 209].

As for the HDACi, vorinostat and panobinostat are potent inducers of γ‐globin and HbF, therefore, are also being considered as a potential therapy for SCD and β‐thalassemia. Vorinostat also has the ability to downregulate α‐globin expression, but the mechanism is not well understood yet [210]. The corresponding Phase 1/2 clinical trial was terminated due to low enrollment (NCT01000155). Among the five adult SCD patients who completed the trial, no convincing induction of HbF was observed [211]. In a small study, patients without hemoglobinopathies experienced a slight increase in HbF following treatment with panobinostat [212]. The Phase 1 clinical trial of panobinostat for the treatment of SCD is currently active but yet recruiting (NCT01245179). Although HDACi have not yet demonstrated satisfactory efficacy and safety in hemoglobinopathies, givinostat shows the ability of HDACi to acetylate HSP90 and disrupt its chaperone function, thereby downregulating JAK2, is particularly appealing for PV treatment [213]. In the interim analysis, which included 51 patients with PV who had previously participated in Phase 1/2 studies and/or received givinostat through compassionate use programs, the overall response rate remained above 80% throughout the mean follow‐up period of 4 years [214]. A randomized, open‐label, multicenter Phase 3 study evaluating the efficacy and safety of givinostat versus HU in patients with high‐risk PV positive for the JAK2V617F mutation is currently ongoing (NCT06093672).

Besides, EPO is one of the most well‐known examples of successful glycoengineering [215]. The addition of glycosylation sites in EPO enhances half‐life and biological activity of EPO. Recombinant human EPO has important therapeutic applications in the treatment of anemia [216].

The development of protein modification agents has achieved gratifying results, with an increasing number of novel drugs entering clinical trials. This progress is largely attributed to the deeper understanding of PTMs. As research progresses, the integration of novel insights into PTMs with innovative therapeutic strategies holds the potential to significantly improve the management of erythroid diseases, offering hope for more effective and safer treatments in the future.

## 6 Challenges and Opportunities of PTM Clinical Translation

Targeting PTMs in drug development represents a rapidly evolving and highly promising field in modern therapeutics. However, the journey of PTMs‐targeted drug development is not without challenges. Given the intricate regulatory networks of PTMs in both physiology and pathology, perturbing the modifying enzymes can simultaneously affect metabolism and trigger PTM crosstalk, ultimately compromising drug safety. Consequently, more precise target selection, site‐specific development, and rational combination of drugs targeting distinct PTM types have emerged as key strategies to overcome current bottlenecks.

In terms of observed outcomes from erythroid‐directed applications and studies, the most critical aspect of drug development is the challenge of drug safety. Some PTM drugs have demonstrated promising therapeutic efficacy in clinical trials but are also associated with potential toxicities. JAK inhibitors could be associated with an extra risk to develop skin cancers [188, 229]. Immune suppression is one of the main risk factors for the development of skin cancer, particularly Merkel cell carcinoma and nonmelanoma skin cancers [230, 231]. The use of JAK inhibitors weakens normal immune surveillance, including reduced clearance of cancer cells and increased susceptibility to infections with certain oncogenic pathogens associated with skin cancer [232]. Activation of PK lowers 2,3‐DPG levels, reducing HbS polymerization and sickling, but potentially impairing oxygen release to tissues [233]. HDAC participation in multiple cellular and physiological functions, limit the achievement of epigenetic pharmacodynamic effect, and thus of HbF induction in vivo [234]. Moreover, the delivery and metabolism of PTM drugs in the human body are also fraught with challenges. For example, UNC0638 showed strong HbF‐inducing activity in ex vivo erythroid cell culture systems, but its in vivo pharmacokinetic properties were proven to lack drug‐like characteristics [235].

Not only do the metabolic changes caused by PTM drugs or adverse reactions due to crosstalk of PTM pathways need to be taken seriously, but also the accessibility and adherence to these drugs cannot be ignored at present. Given that the efficacy of ruxolitinib relies on continuous administration, a prospective multicenter study from Italy found that almost half of the patients reported low adherence to ruxolitinib. The main reason was the difficulty in obtaining the drug and prolonged (>1 year) therapy [236]. While a severe ruxolitinib discontinuation syndrome as observed in myelofibrosis has not been reported in PV, the discontinuation of ruxolitinib in PV usually leads to an increase in Hct values and the reoccurrence of systemic symptoms [237, 238]. Two patients experienced acute hemolysis after abruptly discontinuing a 300‐mg dose of mitapivat, which was associated with a rapid increase in hemoglobin levels. However, the risk of such hemolysis was mitigated when the drug was tapered off gradually [239]. In the clinical translation of PTM therapies, all patients should be fully informed of the risks and be encouraged to enhance medication adherence.

Despite these challenges, the opportunities in PTM‐targeted drug development are vast. Poor selectivity is often the primary cause of severe side effects or toxicity. To address this, improved selective HDACi and covalent inhibitors that target specific protein conformations have been developed to enhance selectivity and reduce off‐target effects [240, 241]. The advent of TPD has revolutionized the field by facilitating the degradation of proteins and enabling the targeting of previously “undruggable” proteins [242, 243]. It has gradually become one of the hot spots of PTM related research in recent years. PROTACs are hetero‐bifunctional molecules that recruit a POI to endogenous E3 ligases, facilitating its ubiquitylation and subsequent proteasomal degradation [244]. The expression of the BCR–ABL protein in HSCs aberrantly activates a series of signaling pathways that promote cell proliferation in CML. Since 2015, over 14 different PROTAC structures targeting the BCR–ABL protein have been reported, showing strong antileukemic activity in vitro and in K562 xenografted mouse models in vivo [245, 246]. Mehta et al. utilized the dTAG PROTAC degradation platform to acutely deplete BCL11A protein, resulting in a potent induction of HBG1/2 (γ‐globin gene) expression [247]. Yan et al. established a human embryonic stem cells line with reversible vimentin degradation (dTAG–VIM–H9) using the PROTAC platform to promote erythroid enucleation [248]. These approaches offer a promising therapeutic strategy for treating erythroid disease. Besides, Ravikumar et al. screened for PRMT5 and EHMT2 inhibitors with better affinity and stability based on molecular dynamics simulations to reactivate γ‐globin gene expression for the treatment of β‐thalassemia, screening for ideal compounds for the development of PTM‐targeted drugs [249, 250]. Nanoparticles and other drug delivery systems can further reduce the side effects of PTM drugs by increasing their serum half‐life and organ specificity [251]. NPs loaded with protein modifiers are being investigated in the fields of autoimmune diseases, cardiovascular diseases, Parkinson's disease, and cancer [252, 253, 254].

In addition to precise PTM drug–target development, the combination of drugs targeting different mechanisms may also benefit patients with erythroid diseases. Merging of ruxolitinib and vorinostat leads to highly potent inhibitors of JAK2 and HDAC6, supported broad cellular antiproliferative potency in hematological cell lines [255, 256]. In a study combining ruxolitinib with pegylated interferon alfa‐2a, the hematologic response rate was very high, with rapid reduction in JAK2V617F variant allele frequency and histologic response, even normalization of bone marrow within 2 years. The relatively low dose of ruxolitinib required may be associated with a lower risk of infection. However, 68% of patients reported peripheral neuropathy, and the long‐term safety of this combination remains to be studied [257]. The combination of decitabine and the LSD1 inhibitor RN‐1 produces a synergistic effect on γ‐globin expression, leading to potent increases in HbF and F‐cells in healthy, nonanemic, and anemic baboons [205].

While many challenges remain, protein modifications offer essential proof‐of‐concept for pursuing the new treatments further. The ongoing advancements in technology and our understanding of PTM biology are paving the way for a new era of precision medicine.

## 7 Conclusion and Prospects

PTMs play critical roles in both physiological and pathological processes. Proteomics captures a dynamic snapshot of an individual's proteome, including the profiles of various protein modifications. This capability to reflect the real‐time actual state of proteins makes proteomics a valuable tool for clinical diagnosis and therapeutics, providing insights into both health and disease [258]. These proteomics data‐integrated databases provide a solid foundation for predicting PTM sites. With the support of various computational methods, prediction tools have been developed to efficiently predict important PTM sites in physiological or pathological processes. However, due to their high false‐positive rates, further model research is still needed to translate these tools into clinical diagnosis [259].

Large‐scale, multiomics profiling of phosphorylation, ubiquitination, methylation, SUMOylation, glycosylation, and acetylation in both physiology and pathology states provides a powerful resource for identifying critical molecular targets and furnishes potential biomarkers for diagnosis, prognosis, and disease stratification. Nevertheless, the pathological specificity of most newly uncovered targets remains uncertain, and modification drift introduced during sample handling—together with the absence of standardized protocols—generates poor interstudy reproducibility, creating a translational gap that must be bridged before laboratory discoveries can be robustly implemented in the clinic. Moreover, the interplay between different PTMs is complex and often involves crosstalk and synergistic functions that are essential for the regulation [260, 261]. Spatiotemporally distinct expression and abundance shifts of the same protein can elicit context‐dependent PTM repatterning, thereby driving divergent pathological outcomes. Therefore, the clinical translation pipeline must also account for the dynamic crosstalk, stoichiometry, and spatial redistribution of diverse PTMs.

Furthermore, while in vitro and in vivo studies have provided foundational knowledge, there is a need for more comprehensive and in‐depth validation, particularly in the context of clinical applicability. PTM‐directed drug development has evolved from modulating modifying enzymes to directly manipulating PTM states, thereby expanding target‐site options and minimizing off‐pathway activity perturbations. As an exemplar of PTM‐driven clinical translation, kinase and HDACi in erythroid disease treatment highlight their efficacy and safety. However, the development of target‐related resistance and the potential for relapse with prolonged use are major concerns. As a leading drug‐development platform that directly targets PTM states, PROTACs leverage the UPS, offer a distinct mechanism for selective protein degradation, potentially reducing resistance associated with small‐molecule inhibitors. While PROTACs are an exciting burgeoning area of drug development, effectively translating these discoveries into clinical applications is an unresolved challenge. Nanodelivery systems enhance therapeutic efficacy by improving pharmacokinetics, extending controlled release, enabling targeted delivery, and facilitating drug release. Their integration with PTM drugs is also an important direction of current research [262].

We have presented a comprehensive review of the current status of PTM studies and outlined potential future clinical translations for this exciting area of research. Deciphering the complex biological functions of proteins still requires advanced technologies development [263]. Ultimately, these technological advancements are expected to improve clinical management and patient outcomes.

## Author Contributions

S. Z., J.L., M.C., and H.H. all contributed to the writing, editing, and figures in the final manuscript. All authors have read and approved the final manuscript.

## Funding

This work was supported by the Major Scientific Research Program for Young and Middle‐aged Health Professionals of Fujian Province (grant no. 2023ZQNZD009), the National Natural Science Foundation of China (grant no. 81970170), Joint Funds for the Innovation of Science and Technology, Fujian Province (grant no. 2023Y9364), Fujian Provincial Natural Science Foundation of China (grant no. 2023J011217, 2025J011152 and 2025J011153).

## Ethics Statement

The authors have nothing to report.

## Conflicts of Interest

The authors declare no conflicts of interest.

## Supporting information




**Figure S1** A simplified model delineates the ontogeny of erythropoiesis and the hemoglobin switch. Erythropoiesis begins in the yolk sac during the embryonic period, then shifts to the liver and spleen, and finally matures and enucleates in the bone marrow before entering the peripheral blood circulation. Globin switching occurs during the same period, transitioning from the expression of embryonic ζ‐globin and ε‐globin, to fetal γ‐globin expression, and finally to the dominance of adult β‐globin expression after birth. A variety of key molecules and transcription factors play important regulatory roles in the stages of erythropoiesis and globin switching to ensure normal red blood cell function. *Abbreviations*: HSC, hematopoietic stem cells; CMP, common myeloid progenitors; MEP, megakaryocytic erythroid progenitors; CFU‐E, colony‐forming unit‐erythroid; BFU‐E, burst‐forming unit‐erythroid; Pro‐E, proerythroblasts; Baso‐E, basophilic erythroblasts; Poly‐E, polychromatic erythroblasts; Ortho‐E, orthochromatic erythroblasts; Retic, reticulocytes; RNA PII, RNA polymerase II.
**Figure S2** Protein phosphorylation involve in the erythropoiesis. The erythroid cytokine receptors c‐Kit and EPOR share overlapping signaling pathways. EPO‐R activation via JAK2 phosphorylation triggers PI3K/AKT, MAPK, and STAT5 pathways, promoting erythroid progenitor cell proliferation, differentiation, and target gene activation. c‐Kit signaling, activated by SCF, promotes precursor cell proliferation and delays differentiation, with downregulation required for terminal differentiation mediated by EPOR/STAT5. MAPK signaling, activated by c‐Kit and EPO, involving ERK2 and ERK1, is crucial for red blood cell maturation.
**Table S1** Ubiquitination modification enzymes and their biological functions associated with erythropoiesis. *Abbreviations*: UPS, ubiquitin–proteasome system; E2, ubiquitin‐conjugating enzymes; E3, ubiquitin ligases; DUBs, deubiquitinating enzymes; WDR26, WD Repeat Domain 26; FBXO11, F‐box protein 11; BAHD1, bromo adjacent homology domain containing 1; PRC, polycomb repressive complex; TRIM, tripartite motif containing; HSC, hematopoietic stem cells; MYSM, Myb Like, SWIRM and MPN Domains 1.

## Data Availability

The authors have nothing to report.
