# Supplementary material for: Post‐translational Modifications in Proteins: Prediction Methods, Biological Functions, and Diseases
Source: MedComm (2020). 2026 Apr 12;7(4):e70729. doi: 10.1002/mco2.70729 (PMC13070201; doi:10.1002/mco2.70729)
Supplement: Supplementary file 1 — Figure S1 A simplified model delineates the ontogeny of erythropoiesis and the hemoglobin switch. Erythropoiesis begins in the yolk sac during the embryonic period, then shifts to the liver and spleen, and finally matures and enucleates in the bone marrow before entering the peripheral blood circulation. Globin switching occurs during the same period, transitioning from the expression of embryonic ζ‐globin and ε‐globin, to fetal γ‐globin expression, and finally to the dominance of adult β‐globin expression after birth. A variety of key molecules and transcription factors play important regulatory roles in the stages of erythropoiesis and globin switching to ensure normal red blood cell function. Abbreviations: HSC, hematopoietic stem cells; CMP, common myeloid progenitors; MEP, megakaryocytic erythroid progenitors; CFU‐E, colony‐forming unit‐erythroid; BFU‐E, burst‐forming unit‐erythroid; Pro‐E, proerythroblasts; Baso‐E, basophilic erythroblasts; Poly‐E, polychromatic erythroblasts; Ortho‐E, orthochromatic erythroblasts; Retic, reticulocytes; RNA PII, RNA polymerase II. Figure S2 Protein phosphorylation involve in the erythropoiesis. The erythroid cytokine receptors c‐Kit and EPOR share overlapping signaling pathways. EPO‐R activation via JAK2 phosphorylation triggers PI3K/AKT, MAPK, and STAT5 pathways, promoting erythroid progenitor cell proliferation, differentiation, and target gene activation. c‐Kit signaling, activated by SCF, promotes precursor cell proliferation and delays differentiation, with downregulation required for terminal differentiation mediated by EPOR/STAT5. MAPK signaling, activated by c‐Kit and EPO, involving ERK2 and ERK1, is crucial for red blood cell maturation. Table S1 Ubiquitination modification enzymes and their biological functions associated with erythropoiesis. Abbreviations: UPS, ubiquitin–proteasome system; E2, ubiquitin‐conjugating enzymes; E3, ubiquitin ligases; DUBs, deubiquitinating enzymes; WDR26, WD Repeat Domain 26; FBXO11, F‐ [file MCO2-7-e70729-s001.docx]

**Post-translational modifications in proteins: prediction methods, biological functions and diseases**

**Shuning Zhang^1, 2, *^, Jingmin Li^1, 2, *^, Meihuan Chen^2^, Hailong Huang^1, 2^**

1.Medical Genetic Diagnosis and Therapy Center of Fujian Maternity and Child Health Hospital, College of Clinical Medicine for Obstetrics & Gynecology and Pediatrics, Fujian Medical University, Fuzhou 350001, People’s Republic of China.

2.Fujian Provincial Key Laboratory for Prenatal Diagnosis and Birth Defect, Fuzhou 350001, People’s Republic of China.

* These authors contributed equally to this work.

**Correspondence author:**

Hailong Huang

e-mail: [huanghailong@fjmu.edu.cn](mailto:huanghailong@fjmu.edu.cn)

ORCID: <https://orcid.org/0000-0003-2924-399X>

Institution and address: Medical Genetic Diagnosis and Therapy Center of Fujian Maternity and Child Health Hospital, Fujian Provincial Key Laboratory for Prenatal Diagnosis and Birth Defect, 18 Daoshan Road, Fuzhou, People’s Republic of China

Telephone: +86 13509366432

Meihuan Chen

e-mail: chenmeihuan@fjmu.edu.cn

Institution and address: Medical Genetic Diagnosis and Therapy Center of Fujian Maternity and Child Health Hospital, Fujian Provincial Key Laboratory for Prenatal Diagnosis and Birth Defect, 18 Daoshan Road, Fuzhou, People’s Republic of China

**
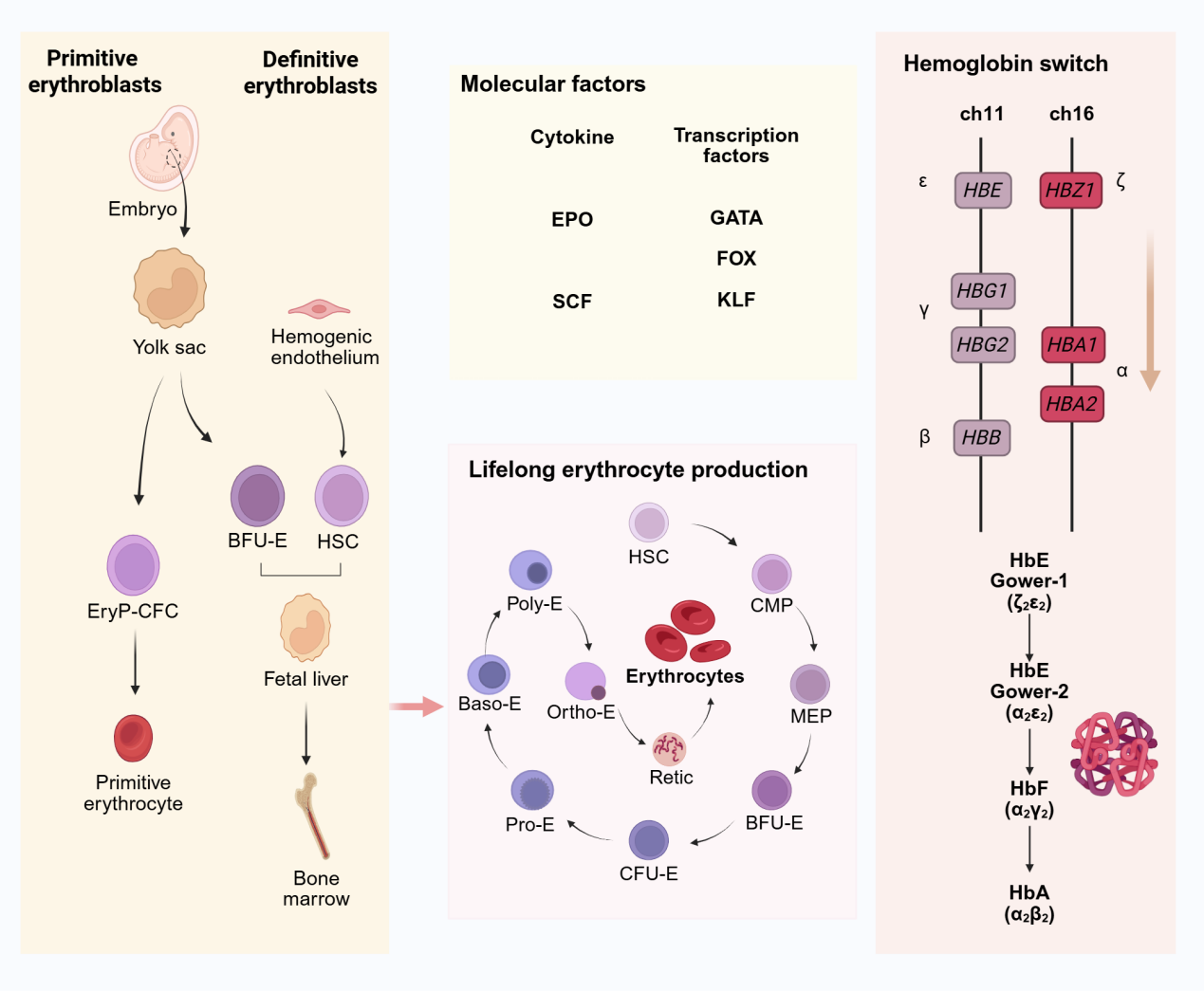
Figure S1. A simplified model delineates the ontogeny of erythropoiesis and the hemoglobin switch**

Erythropoiesis begins in the yolk sac during the embryonic period, then shifts to the liver and spleen, and finally matures and enucleates in the bone marrow before entering the peripheral blood circulation. Globin switching occurs during the same period, transitioning from the expression of embryonic ζ-globin and ε-globin, to fetal γ-globin expression, and finally to the dominance of adult β-globin expression after birth. A variety of key molecules and transcription factors play important regulatory roles in the stages of erythropoiesis and globin switching to ensure normal red blood cell function.

**HSC** —hematopoietic stem cells, **CMP** —common myeloid progenitors，**MEP** —megakaryocytic erythroid progenitors，**CFU-E** —colony-forming unit-erythroid, **BFU-E** —burst-forming unit-erythroid, **Pro-E** —proerythroblasts, **Baso-E** —basophilic erythroblasts, **Poly-E** —polychromatic erythroblasts, **Ortho-E** —orthochromatic erythroblasts, **Retic** —reticulocytes, **RNA PⅡ**—RNA polymerase II

**
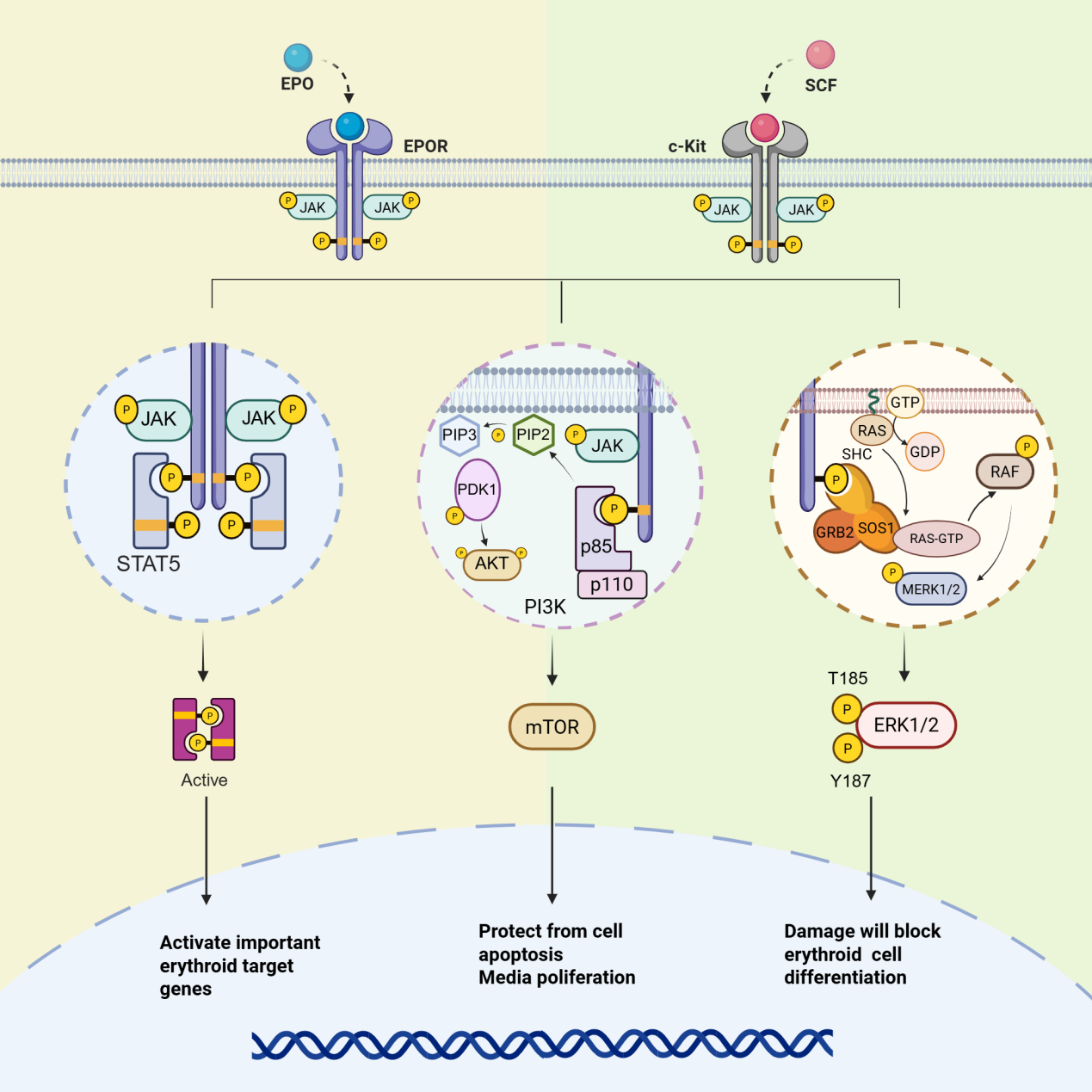
Figure S2. Protein phosphorylation involve in the erythropoiesis**

The erythroid cytokine receptors c-Kit and EPOR share overlapping signaling pathways. EPO-R activation via JAK2 phosphorylation triggers PI3K/AKT, MAPK, and STAT5 pathways, promoting erythroid progenitor cell proliferation, differentiation, and target gene activation. c-Kit signaling, activated by SCF, promotes precursor cell proliferation and delays differentiation, with downregulation required for terminal differentiation mediated by EPOR/STAT5. MAPK signaling, activated by c-Kit and EPO, involving ERK2 and ERK1, is crucial for red blood cell maturation.

| **Enzyme** |  | **Biological functions** |
| --- | --- | --- |
| **E2/E3** | UBE2O | Targeting ribosomal proteins and excess α-globin |
|  | UBE2H | Participating enucleation |
|  | FBXO11 | Degradation of BAHD1 to relieve PRC2-mediated repression |
|  | TRIM58 | Ubiquitinating dynein |
|  | TRIM10/HERF1 | Regulating globin gene regulation |
|  | TRIM28 | Regulating globin gene regulation |
|  | MDM2-MDM4 | Regulating apoptosis |
|  | WDR26 | Regulating the polyubiquitination of a fraction of nuclear proteins, nuclear opening |
| **DUBs** | USP3 | Targeting histone H2A, preserving HSC self-renewal, and repopulation potential |
|  | USP7 | Stabilizing the GATA1 protein |
|  | USP15 | Stabilizing of HSC self-renewal and DNA repair factor |
|  | USP50 | Stabilizing the Ku70 protein |
|  | MYSM1 | Antagonizing PRC1-mediated histone ubiquitination and transcriptional repression |

**Table S1. Ubiquitination modification enzymes and their biological functions associated with erythropoiesis**

**UPS**— ubiquitin-proteasome system, **E2**—ubiquitin-conjugating enzymes, **E3**—ubiquitin ligases, **DUBs**—deubiquitinating enzymes, **WDR26**—WD Repeat Domain 26, **FBXO11**—F-box protein 11, **BAHD1**—bromo adjacent homology domain **c**ontaining 1, **PRC**—polycomb repressive complex, **TRIM**—tripartite motif containing, **HSC**—hematopoietic stem cells, **MYSM**—Myb Like, SWIRM And MPN Domains 1
